# Selective depletion of tumor-infiltrating regulatory T cells with BAY 3375968, a novel Fc-optimized anti-CCR8 antibody

**DOI:** 10.1007/s10238-024-01362-8

**Published:** 2024-06-10

**Authors:** Helge G. Roider, Sabine Hoff, Su-Yi Tseng, Sandra Berndt, Mark Trautwein, Katharina Filarsky, Uwe Gritzan, Jordi Camps, Wiebke Maria Nadler, Joanna Grudzinska-Goebel, Philipp Ellinger, Theresa Pesch, Chai Fen Soon, Marcel Geyer, Katja Gluske, Beatrix Stelte-Ludwig, Mátyás Gorjánácz

**Affiliations:** 1 Bayer AG, Pharmaceuticals, Berlin, Germany; 2 Bayer AG, Pharmaceuticals, San Francisco, USA; 3 Bayer AG, Pharmaceuticals, Wuppertal, Germany; 4 Current address: Roche Diagnostics GmbH, Penzberg, Germany; 5 Current address: Memorial Sloan Kettering Cancer Center, New York, USA; 6 Current address: Vincerx Pharma, Monheim am Rhein, Germany

**Keywords:** Chemokine receptor 8, Regulatory T cells, Monoclonal antibody, Cancer immunotherapy, Immunosuppression

## Abstract

Regulatory T cells (Tregs) are known to facilitate tumor progression by suppressing CD8+ T cells within the tumor microenvironment (TME), thereby also hampering the effectiveness of immune checkpoint inhibitors (ICIs). While systemic depletion of Tregs can enhance antitumor immunity, it also triggers undesirable autoimmune responses. Therefore, there is a need for therapeutic agents that selectively target Tregs within the TME without affecting systemic Tregs. In this study, as shown also by others, the chemokine (C–C motif) receptor 8 (CCR8) was found to be predominantly expressed on Tregs within the TME of both humans and mice, representing a unique target for selective depletion of tumor-residing Tregs. Based on this, we developed BAY 3375968, a novel anti-human CCR8 antibody, along with respective surrogate anti-mouse CCR8 antibodies, and demonstrated their in vitro mode-of-action through induction of potent antibody-dependent cellular cytotoxicity (ADCC) and phagocytosis (ADCP) activities. In vivo, anti-mouse CCR8 antibodies effectively depleted Tregs within the TME primarily via ADCP, leading to increased CD8+ T cell infiltration and subsequent tumor growth inhibition across various cancer models. This monotherapeutic efficacy was significantly enhanced in combination with ICIs. Collectively, these findings suggest that CCR8 targeting represents a promising strategy for Treg depletion in cancer therapies. BAY 3375968 is currently under investigation in a Phase I clinical trial (NCT05537740).

**Supplementary Information:**

The online version contains supplementary material available at 10.1007/s10238-024-01362-8.

## Introduction

Immune checkpoint inhibitors (ICIs) have revolutionized the treatment of cancer by increasing survival and achieving durable therapeutic responses for some cancer patients. However, primary and acquired resistance to ICIs frequently occurs [1] with regulatory T cells (Tregs) identified as one of the key resistance mechanisms [2]. Tregs are CD4+ T cells characterized by expression of lineage-specific transcription factor Forkhead box P3 (FoxP3). They suppress excessive immune responses, thereby maintaining immune homeostasis and tolerance to self-antigens. Reduced systemic Treg activity leads to loss of immune suppression, potentially promoting diverse autoimmune disorders [3–5]. Conversely, Tregs can also compromise immuno-surveillance against cancer, and high abundance of Tregs relative to CD8+ effector T cells in the tumor microenvironment (TME) is associated with poor prognosis across multiple cancer indications [5–9]. Mechanisms utilized by Tregs include secretion of anti-inflammatory cytokines such as interleukin (IL)-10 or transforming growth factor-beta (TGF-β), sequestration of IL-2 via high expression of its high-affinity receptor CD25, or high expression of inhibitory surface receptors such as cytotoxic T-lymphocyte-associated protein 4 (CTLA-4) [10, 11]. Depleting Tregs has therefore been proposed as a new way to elicit anticancer immune reactions and to overcome resistance to ICIs.

Target cell depleting antibodies function by binding to specific fragment crystallizable gamma receptors (FcγRs) expressed on effector cells like NK cells and macrophages, or to complement component 1q (C1q), thereby inducing antibody-dependent cellular cytotoxicity (ADCC), antibody-dependent cellular phagocytosis (ADCP), or complement-dependent cytotoxicity (CDC) [12–14]. In cancer therapies, most depleting antibodies belong to immunoglobulin 1 (IgG1) subclass and often rely on ADCC to eliminate cancer cells [14]. The effectiveness of this effector function is influenced by the N-linked glycosylation in the Fc region of the antibody. Specifically, the absence of the core fucose on the Fc N-glycan has been shown to enhance the binding affinity of IgG1s for FcγRIIIA expressed on NK cell, thereby augmenting ADCC activity [15]. ADCP is an emerging mechanism of action for depleting antibodies and has been proposed as an additional effector function for several U.S. Food and Drug Administration (FDA)-approved monoclonal antibodies like rituximab (chimeric anti-CD20 of IgG1 isotype) and trastuzumab (humanized anti-HER2 of IgG1 isotype) used for treatments of non-Hodgkin lymphoma (NHL) and metastatic breast cancer or gastric cancer patients, respectively [16–22].

Current clinical strategies for depleting Tregs rely on cell surface targets that are expressed on both peripheral Tregs and CD8+ T effector cells. Ipilimumab, a fully human anti-human CTLA-4 antibody of IgG1 isotype, was the first approved ICI that augments priming and activation of CD8+ effector T cells via blocking CTLA-4 signaling [10, 23, 24]. Although this activity was regarded as the primary mechanism of ipilimumab, growing evidence suggests that depletion of Tregs may also play an important role. In preclinical models for example the efficacy of anti-CTLA-4 antibodies is profoundly amplified by FcγR-mediated depletion of tumor-infiltrating Tregs [25–28], and depletion of Tregs from human tumors was also observed when tumor biopsies were taken shortly after ipilimumab treatments [29, 30]. However, since CTLA-4 is not expressed tumor-specifically, severe immune-related adverse events limit its application [11, 31]. Other Treg-targeting approaches include therapeutic antibodies targeting CD25 and chemokine (C–C motif) receptor 4 (CCR4). However, although these antibodies efficiently deplete peripheral Tregs and may reduce tumor-infiltrating Tregs, durable clinical responses in patients with solid tumors have not been observed [32–37]. This might be due, at least in part, to co-depletion of recently activated tumor-reactive CD8+ T cells, thus limiting clinical responses [38]. Therefore, there is a high need for new safe and effective agents that would specifically deplete tumor-infiltrating Tregs, while sparing both peripheral Tregs and effector T cells.

Chemokine (C–C motif) receptor 8 (CCR8) is predominantly expressed on activated Tregs marking the most suppressive and proliferative Treg population residing in the TME [39–45]. CCR8+ Tregs are associated with high tumor grade and poor overall survival across many tumor types [39, 46, 47]. Consequently, unlike other Treg-directed approaches, targeting CCR8 offers the opportunity to specifically deplete intratumoral Tregs without impacting peripheral Tregs or other immune cells. This makes CCR8 a prime target in cancer immunotherapy, with several different assets in preclinical development or already in Phase I clinical trials.

Here, we describe the development and characterization of BAY 3375968, a novel fully human afucosylated monoclonal IgG1 anti-human CCR8 antibody for treatment of human solid cancers. We show that CCR8 expression is restricted to activated Tregs that mainly reside within the TME and demonstrate that BAY 3375968 efficiently depletes CCR8+ cells in an FcγR-dependent manner in vitro. Due to low sequence homology, mouse surrogate antibodies for BAY 3375968 were co-developed to describe the in vivo pharmacodynamic (PD) profile of CCR8+ Treg depletion. Further to these encouraging preclinical data, a Phase I clinical trial of BAY 3375968 is currently ongoing in patients with advanced solid tumors (NCT05537740).

## Materials and methods

### Antibodies

The antibodies used in the study were generated in-house at Bayer AG or purchased from providers as described in Supplementary Table 1. Generation of anti-CCR8 antibodies is described in the Supplementary Methods section.

### Single-cell *CCR8* mRNA expression in human tumors

To characterize the expression pattern of *CCR8* across human immune cells from normal and tumor tissue, we used the collection of public single-cell RNA-Seq datasets from the IMMUcan SingleCell RNA-Seq Database [48]. Processed single-cell objects were downloaded in h5ad format from https://immucanscdb.vital-it.ch/. Only studies focused on immune cells and samples covering both tumor and matching normal tissue were selected. Studies which contained less than 20 Tregs were removed leaving the following datasets for analysis: GSE120575 (melanoma, Smart-Seq2 platform), GSE123139 (melanoma, MARS-seq platform), GSE140228 (hepatocellular carcinoma, Smart-Seq2 and 10x), GSE146771 (colorectal carcinoma, Smart-Seq2 and 10x), GSE164522 (colorectal carcinoma, Smart-Seq2), GSE139555 (NSCLC, colorectal and endometrial carcinoma, 10x), and GSE114725 (breast cancer, inDrop platform). Single cells were grouped via Louvain graph-based clustering implemented in the *Seurat* package (version 4). Tregs were defined in each dataset as the cluster of cells most highly expressing the Treg lineage marker *FoxP3*. Activated Treg clusters were defined to further express high levels of the common T cell activation marker *4-1BB* (*TNFRSF9). Z-*scaled average expression of *CCR8* across all immune cell types from normal and tumor tissue was visualized using a heatmap. Differential gene expression analysis between activated and resting Tregs as well as between Tregs and all other immune cell types was performed using the function FindAllMarkers from the Seurat package with default parameters (except for applying a pseudocount of 0.1). Resulting log2 fold changes from each comparison were visualized for all genes using a scatter plot.

### Bulk mRNA expression analysis of antibody-treated mouse tumors

Gene expression profile of murine PANC02, Hepa1-6, MBT2, MC38, H22, and CT26 syngeneic tumors treated with either anti-mouse CCR8 mIgG2a and hIgG1 antibody variants or with the respective isotype controls was determined via RNA-seq. To this end tumor samples were collected one day after the last antibody treatment (BIW for 3 weeks), flash frozen in liquid nitrogen and homogenized. RNA was extracted from 30 mg of frozen tissue using Qiagen RNeasy Mini Kit (Cat#74,106). RNA quality was assessed via 2100 Bioanalyser (Agilent) and quantified using NanoDrop (Thermo Scientific). Only high-quality RNA samples (OD260/280 = 1.8 ~ 2.2, OD260/230 ≥ 2.0, RIN ≥ 7, > 500 ng) were used to construct sequencing libraries.

PolyA mRNA was purified from total RNA using oligo-dT-attached magnetic beads and then fragmented in fragmentation buffer. Libraries were constructed using random first strand primers and according to Illumina’s library construction protocol. After library construction, Qubit 2.0 fluorometer dsDNA HS Assay (Thermo Fisher Scientific) was used to quantify concentration of the resulting sequencing libraries, while the size distribution was analyzed using Agilent BioAnalyzer 2100 (Agilent). After library validation, Illumina cBOT cluster generation system with HiSeq PE Cluster Kits (Illumina) was used to generate clusters. Paired-end sequencing was performed using an Illumina system following Illumina-provided protocols for 2 × 150 paired-end sequencing. Reads were mapped to mouse genome version mm10 and gene expression was quantified using estimated counts provided by the RSEM algorithm (https://doi.org/10.1186/1471-2105-12-323). Counts were normalized for library sizes using estimated size factors provided by DESeq2 (https://doi.org/10.1186/s13059-014-0550-8). Log2 expression levels in anti-CCR8 and isotype-treated tumors were visualized using boxplots.

### Cellular binding affinity of anti-CCR8 antibodies to mouse or human CCR8

The in vitro binding of the anti-mouse CCR8 antibodies (hIgG1, Fc-silenced N297A-aglycosylated hIgG1, or chimerized to mIgG2a) and the respective non-binding isotype control antibodies to mouse CCR8 was studied using mouse CCR8-transfected HEK293 cells and HEK293 cells transfected with empty vector (InSCREENex GmbH). Similarly, the in vitro binding of the anti-human CCR8 antibodies (BAY 3375968, BAY 3353497, or fully Fc-silenced LALA-aglycosylated L234A, L235A, N297A variant) and the respective non-binding hIgG1 isotype control antibodies were studied using human CCR8-transfected CHO or HEK293 cells and compared with CHO and HEK293 cells transfected with empty vector (InSCREENex GmbH). Briefly, cells were stained with anti-CCR8 antibodies as primary antibody followed by R-Phycoerythrin conjugated secondary antibodies and analyzed using a BD FACSCanto II™ flow cytometry system (BD Biosciences). Detailed information on the protocols can be found in the Supplementary Methods section.

### In vitro ADCC activity of anti-CCR8 antibodies

The antibody-dependent cellular cytotoxicity (ADCC) activities of the anti-mouse CCR8 antibodies (hIgG1, its Fc-silenced variant N297A-aglycosylated hIgG1, or chimerized to mIgG2a) were determined using murine CCR8-transfected HEK293 cells as target cells and the non-binding isotype control antibodies as controls. Primary mouse NK cells, pre-activated with rmIL-15 for 24 h, were used as effector cells. Target cells were stained with IncuCyte^®^ Cytolight Rapid Red Dye (Sartorius) and incubated in co-culture with effector cells in the presence of Incucyte^®^ Caspase-3/7 Green Dye (Sartorius) in the medium to determine living and apoptotic cells, respectively. Therapeutic antibodies were added at various concentrations to the co-culture of effector (E) to target (T) cells at E:T ratio of 10:1 and co-incubated for 20 h. The ADCC potential of the anti-mouse antibodies was evaluated by measuring the apoptosis induction using the Incucyte® S3 Live-Cell Analysis System (Sartorius).

The ADCC activities of anti-human CCR8 afucosylated hIgG1 antibody BAY 3375968, its conventionally glycosylated variant BAY 3353497, its LALA-aglycosylated Fc-silenced variant, and the respective non-binding isotype controls were determined using different human ADCC settings. To this end, primary human Tregs or human CCR8 expressing HEK293 cells were used as target cells. Human Tregs were isolated from PBMCs obtained from healthy donors and stimulated by CD3/CD28 MACSiBead particles (130-091-441, T Cell Activation/Expansion Kit, Miltenyi Biotech) and rIL-2 (R&D Systems). Their CCR8 expression levels were confirmed by flow cytometry. The effector cells were either primary human NK cells or NK92v cells (NantKwest). Therapeutic antibodies were added at various concentrations to the co-culture of effector and target cells at E:T ratio of 4:1 and incubated for 2 or 4 h. The ADCC potential of the anti-human CCR8 antibodies was evaluated by measuring the apoptosis induction by either using a CytoTox-Glo assay (Promega) or a Cytotoxicity Detection Kit (LDH, Roche). Mogamulizumab, an anti-human CCR4 antibody (Kyorin Pharmaceuticals Co), was used as reference. In general, the percentage of cytotoxicity was calculated in relation to the no-antibody control using the formula: ADCC % = [(experimental value—no-antibody control) / (maximal lysis of target cells—spontaneous lysis of target cells)] × 100%. Detailed information on these newly established protocols can be found in the Supplementary Methods section.

### In vitro ADCP activity of anti-CCR8 antibodies 

The antibody-dependent cellular phagocytosis (ADCP) activities of the anti-mouse CCR8 antibodies (hIgG1, its Fc-silenced variant N297A-aglycosylated hIgG1, or chimerized to mIgG2a) were determined using murine CCR8-transfected HEK293 cells as target cells and the non-binding isotype control antibodies as controls. The effector cells were primary mouse bone marrow-derived M2 macrophages. Briefly, bone marrow cells were isolated and differentiated in medium containing 20 ng/mL macrophage colony-stimulating factor (M-CSF; BioLegend, #576,406), and on day 6 cells were polarized into M2 macrophages with 20 ng/mL M-CSF, 50 ng/mL rmIL-4 (BioLegend, #574,306) and 50 ng/ml rmIL-13 (BioLegend, #575,906) for 1 day. Target cells were stained with IncuCyte® Cytolight Rapid Green Dye (Sartorius) and with the pH sensitive IncuCyte® pHrodo™ Red Cell Labeling Dye (Sartorius) for visualization of living and phagocytic cells, respectively. Therapeutic antibodies were added at various concentrations to the co-culture of effector (E) to target (T) cells at E:T ratio of 4:1 and were incubated for 4 h. The ADCP potential of the anti-mouse CCR8 antibodies was determined by fluorescence scanning with Incucyte® S3 Live-Cell Analysis System.

The ADCP activities of anti-human CCR8 afucosylated hIgG1 antibody BAY 3375968, its conventionally glycosylated variant BAY 3353497, its LALA-aglycosylated Fc-silenced variant, and the respective non-binding isotype controls were determined using different human ADCP settings. To this end, either primary human Tregs or human CCR8 expressing HEK293 cells were used as target cells. Human Tregs were isolated from PBMCs obtained from healthy donors and stimulated by CD3/CD28 MACSiBead particles (130-091-441, T Cell Activation/Expansion Kit, Miltenyi Biotech) and rIL-2 (R&D Systems). CCR8 expression levels were confirmed by flow cytometry. In all ADCP experiments the effector cells were human blood monocyte-derived M2 macrophages obtained via cultivating monocytes for 5 days in the differentiation mediums supplemented with 50 ng/mL M-CSF (BioLegend, #574,806), and polarized with 10 ng/mL recombinant human IL-10 (BioLegend, #571,004) for 2 days. In the experiments with human Tregs, effector cells and CFSE-labeled target cells were co-cultured at E:T ratio of 4:1 for 4 h in the presence of various antibody concentrations. Phagocytosis was subsequently determined by flow cytometry as percentage of CFSE+ CD206+ double-positive macrophages. Mogamulizumab, an anti-human CCR4 antibody (Kyorin Pharmaceuticals Co), was used as a reference. In the experiments with human CCR8 expressing HEK293 target cells, target cells were labeled with IncuCyte® Cytolight Rapid Green Dye (Sartorius) and with IncuCyte® pHrodo™ Red Cell Labeling Dye (Sartorius) and were co-cultured with M2 macrophages at E:T ratio of 10:1 for 24 h in the presence of antibodies at various concentrations. The ADCP potential of the anti-human CCR8 antibodies was determined by fluorescence scanning with Incucyte® S3 Live-Cell Analysis System. In general, we determined as real-time ADCP, the % of target cells that are being phagocytosed at the given time point, and as cumulative ADCP, the % of target cells that were removed by phagocytosis until the given time point, both in relation to no-antibody controls. Detailed information on these newly established protocols can be found in the Supplementary Methods section.

### In vitro CDC activity of anti-human CCR8 antibodies 

Treg cells were isolated from human PBMCs of healthy donors using magnetic-based EasySep™ Human CD4+ CD127^low^ CD25+ Regulatory T Cell Isolation Kit (Miltenyi Biotec) and subsequently expanded and activated in vitro by treatment with IL-2 (130–097-745, Miltenyi Biotec) and CD3/CD28 antibodies containing TransACT (130–128-758, Miltenyi Biotec) to induce CCR8 expression. Flow cytometry analysis verified that more than 80% of CD4+ CD25+ FoxP3+ Treg cells expressed CCR8. These cells were then exposed to CDC-qualified human complement serum (Quidel) and various antibodies in a dose-response manner at 37 °C for 3 h. Cells were stained with 7-AAD and cell death induced by CDC is indicated as percentage of 7-AAD-positive events, referencing to negative controls wells with either Treg cells only or Treg cells with 10% serum. Detailed information on the protocols can be found in the Supplementary Methods section.

### Ex vivo analysis of immune cells in mouse tumors and spleens

Determination of Tregs, CD8+ T cells, and other immune cell sub-types in mouse tumors or spleens was performed by flow cytometry. The harvested tumor or spleen samples were dissociated by tissue dissociation with gentleMACS^TM^ Octo Dissociator (Miltenyi Biotec) and mouse Tumor Dissociation Kit (Miltenyi Biotec). Single-cell suspensions were transferred through a 70-µm strainer and cells were resuspended in FACS buffer (3% FCS in PBS). LIVE/DEAD^TM^ Fixable Violet Dead Cell Stain Kit (Invitrogen, #L34955) was used for detecting dead cells. Fc-blocking reagent (CD16/CD32 monoclonal antibody, eBioscience #14-0161-82) was added to ensure that only antigen-specific binding was observed. Cells were stained with APC-Cy7-labeled anti-CD45 antibody (Ab) (BioLegend, #103,116) or BUV421-labeled anti-CD45 Ab (BioLegend, #103,133), BUV737-labeled anti-CD4 Ab, (BD Biosciences, #564,933 or #48–0032-80), FITC-labeled anti-CD25 Ab (BioLegend, #102,017) or Alexa Fluor 488-labeled anti-CD25 Ab (eBioscience, #53-0251-82), PE-labeled anti-FoxP3 Ab (eBioscience, #12-5773-80B) or BV421-labeled anti-FoxP3 Ab (BD Horizon, #562,996), PE-labeled anti-CCR8 Ab (BioLegend, #150,312) or APC-labeled anti-CCR8 Ab (BioLegend, #150,310), BUV395-labeled anti-CD8 Ab (BD Horizon, #563,786) or PE-Cy7-labeled anti-CD8 Ab (eBioscience, #25-0081-82), PECY7-labeled anti-CD44 Ab (Biolegend, #103,030), and APC-labeled anti-CD62L Ab (BioLegend, #104,412), and the number of immune cells expressing the respective marker combinations was determined by fluorescence-activated cell sorting (FACS) using FACSCanto^TM^ (BD Biosciences, San Diego, CA, USA).

### Animal studies to investigate the in vivo activity of anti-mouse CCR8 antibodies 

The antitumor efficacy, mode-of-action, pharmacokinetic (PK)/pharmacodynamic (PD) relationship, and ICI combination potential of anti-CCR8 therapy were assessed in vivo using different syngeneic mouse tumor models. Tumor responses were assessed by determination of the tumor volume (*V* = 0.5 × L × W^2^, where *L* and *W* refer to tumor length and width, respectively) using a caliper, and animal body weight changes were measured 2–3 times weekly. T/C (Treatment/Control) ratios were calculated using the mean tumor volumes, and treatment responses were defined using modified RECIST criteria [49]. The mouse experiments were performed under the national animal welfare laws and approved by the local authorities.

For efficacy and mode-of-action study, female BALBc mice (9-week-old, Charles River) were inoculated subcutaneously (s.c.) with 1 × 10^5^ CT26 murine colon cancer cells on day 0. On day 7, at an average tumor size of 100 mm^3^, i.p. (intraperitoneal) administration of non-binding isotype control antibodies or anti-mouse CCR8 (hIgG1, its Fc-silenced variant N297A-aglycosylated hIgG1, or chimerized to mIgG2a) antibodies started.

For in vivo investigation of antibody dose response, female BALBc mice (9-week-old, Charles River) were inoculated s.c. with 5 × 10^5^ EMT6 murine mammary carcinoma cells, and on day 7, at an average tumor size of 85 mm^3^, mice were treated i.p. with 0.01, 0.1, 1, or 10 mg/kg of anti-mouse CCR8 mIgG2a antibody and observed until day 19.

For studying the PK/PD relationship, female BALBc mice (12-week-old, Charles River) were inoculated s.c. with 5 × 10^5^ EMT6 murine mammary carcinoma cells, and on day 8, at an average tumor size of 35 mm^2^, mice were treated i.p. with a single-dose of non-binding isotype control (4 mg/kg) or with anti-mouse CCR8 mIgG2a antibody (0.25, 1, or 4 mg/kg). Mice (*n* = 5/group/time point) were sacrificed at time points 24, 48, 120, 192, and 336 h after the single-dose administration of the compounds or when the tumors reached the predetermined size of 225 mm^2^. Tumors were collected for analysis of intratumoral Tregs, and blood for antibody exposure analysis.

For combination studies, anti-mouse CCR8 mIgG2a antibody was combined with immune checkpoint inhibitors anti-PD-L1 or anti-PD-1 using MC38 (also referred as C38) murine colon carcinoma or MBT-2 and MB49 murine bladder carcinoma models. In the anti-PD-L1 combination study, female C57BL/6N mice (6–8-week-old, Charles River) were inoculated s.c. with 5 × 10^5^ MC38 / C38 cells on day 0. On day 10, at an average tumor size of 60 mm^3^, i.p. treatments with the non-binding isotype control antibodies, anti-mouse CCR8 mIgG2a antibody, anti-PD-L1, or with combination of the anti-mouse CCR8 mIgG2a antibody and anti-PD-L1 antibody started. After five treatments, on day 24, treatments were completed, and mice were observed for tumor regrowth or tumor regression until day 94. In anti-PD-1 combination studies, we used two syngeneic mouse tumor models. Female C3H/HeJ mice (6–8 weeks old, Vital River Laboratories) were inoculated s.c. with 4 × 10^6^ MBT2 cells, and on day 10, at an average tumor size of 100 mm^3^, i.p. treatment with isotype controls, anti-mouse CCR8 mIgG2a antibody, anti-PD-1 antibody, or the combination of the anti-mouse CCR8 mIgG2a antibody and the anti-PD-1 antibody started. Similarly, female C57BL/6N mice (7 weeks old, Charles River) were inoculated s.c. with 2 × 10^5^ MB49 cells and i.p. treatments started on day 4, at an average tumor size of 40 mm^3^.

### Long-term in vivo efficacy of anti-mouse CCR8 antibody 

The long-term in vivo antitumor efficacy of the anti-mouse CCR8 antibody hIgG1 was studied in three subsequent studies using the CT26 model. In the first phase, female BALBc mice (9-week-old, Charles River) were inoculated s.c. with 1 × 10^5^ CT26 cells in the right flank on day 0. On day 11 at an average tumor size of 80 mm^3^, i.p. treatments with non-binding isotype control or the anti-mouse CCR8 hIgG1 antibody started (*n* = 10). Mice were sacrificed individually 22–77 days post tumor cell inoculation when the tumor volume reached 1,400 mm^3^.

In the second phase, approximately 10 weeks after the original tumor cell inoculation (day 69; 46 days after the last treatment), tumor-free mice (*n* = 4) were re-challenged by a new s.c. injection of 1 × 10^5^ CT26 cells on the contra-lateral flank. The same number of CT26 cells were inoculated into age-matched naïve mice (*n* = 8), which were used as controls. The tumor growth on both the original tumor inoculation site and the new site was monitored. Three weeks after the re-challenge (day 90), mice were sacrificed, and their spleens were collected for splenocyte isolation and analysis of CD8-positive T cell populations**.**

In the third phase, the splenocytes collected in the second phase were adoptively transferred into naïve BALBc mice by an intravenous (i.v.) injection on day 90 and mice were allocated to groups based on the origin of splenocytes (*n* = 10 mice/group). In addition, a control group without splenocyte transfer was included. Next day (day 91), mice were inoculated s.c. with 1 × 10^5^ CT26 cells for another re-challenge. After a follow-up period of 19 days, mice were sacrificed.

## Results

### CCR8 expression is predominant on activated tumor-infiltrating regulatory T cells

**Fig. 1:**
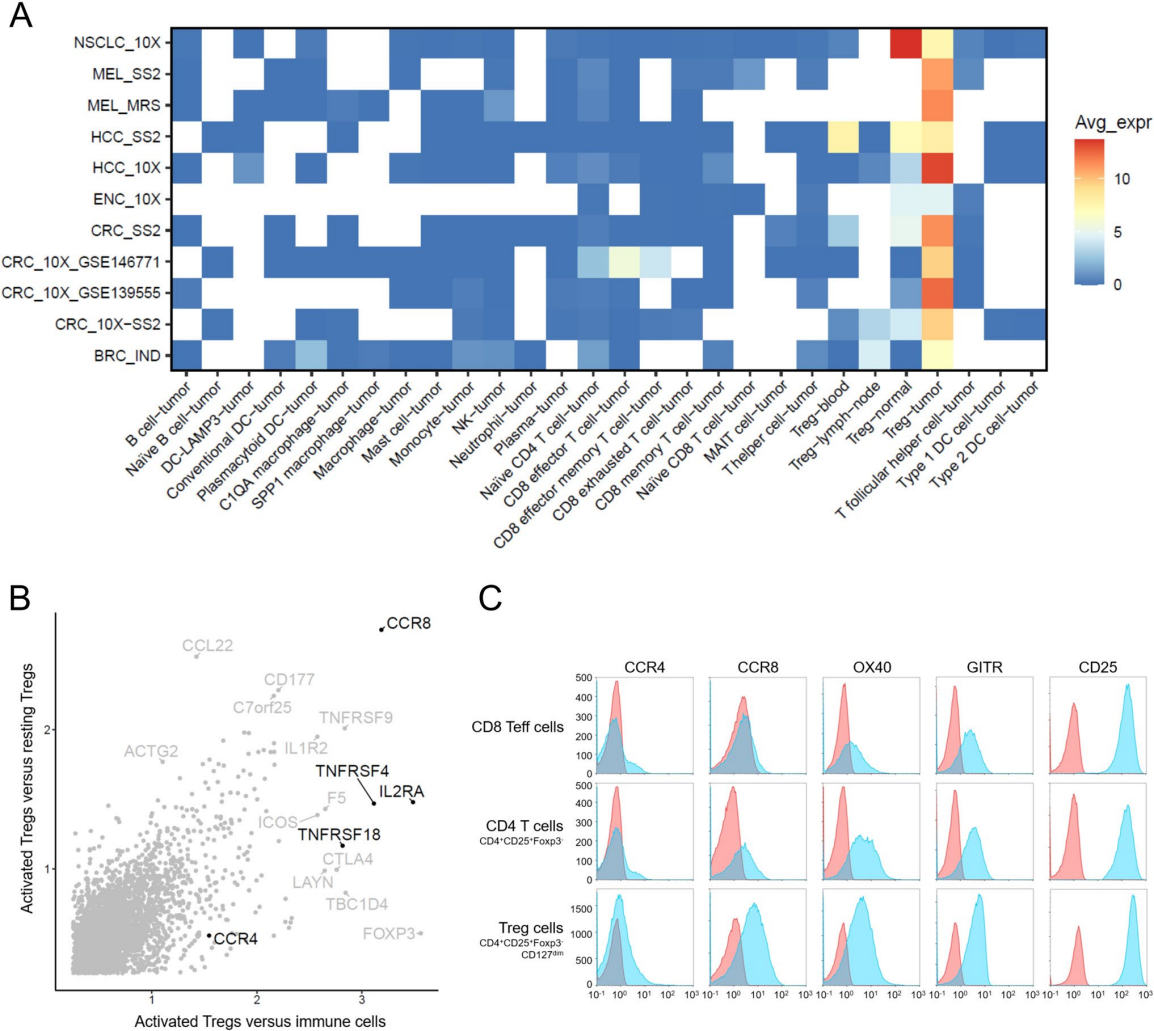
CCR8 is predominantly expressed in activated tumor-infiltrating Tregs. **A** Expression of CCR8 across a variety of human cancer and immune cell types as determined by gene expression analysis of single-cell RNA-Seq datasets. Rows indicate cancer data sets and corresponding indications and columns denote immune cell types. Blue to red colors indicate the average expression of CCR8 across all cells assigned to a cell type. White squares indicate the absence of a cell type from a given dataset. NSCLC, non-small cell lung carcinoma; MEL, melanoma; HCC, hepatocellular carcinoma; ENC, endometrial carcinoma; CRC, colorectal cancer BRC, breast cancer; DC, dendritic cell; MAIT, mucosal-associated invariant T cell; NK, natural killer cell. **B** Differential gene expression between activated Tregs and resting Tregs or other immune cell types. X- and Y-axes represent linear expression fold changes. Highlighted genes were further analyzed in C by flow cytometry. **C** Comparison of surface expression of CCR8 protein and other common Treg receptors as measured by flow cytometry. Human PBMCs were activated using anti-CD3/anti-CD28 stimulation for 6 days and gated for Treg (CD4+CD25+FoxP3+CD127^low^), as well as CD8+ and CD4+ effector T cell populations (*n* = 2 donors). In contrast to the other markers, CCR8 expression was largely limited to the activated Treg population

To identify cell surface markers specific for tumor-residing regulatory T cells we compared the mRNA expression of the Treg lineage marker *FoxP3* across the different TCGA (The Cancer Genome Atlas) tumor cohorts with the mRNA expression profile of all genes in the human genome. We found *CCR8* to be among the best correlated genes across all indications pointing to co-expression of these genes in tumor-residing Tregs (data not shown). An evaluation of eleven single-cell RNA-Seq datasets obtained from the IMMUcan scDB database [48] covering immune cells isolated from different human cancer types including non-small cell lung carcinoma (NSCLC), melanoma (MEL), hepatocellular carcinoma (HCC), endometrial carcinoma (ENC), colorectal carcinoma (CRC), and breast cancer (BRC), as well as respective adjacent normal tissues, confirmed predominant gene expression of *CCR8* in tumor-residing Tregs (Fig. 1A). Expression was typically lower in Tregs isolated from adjacent normal tissue or peripheral blood. No expression of *CCR8* was observed in other cell types. When comparing the expression of *CCR8* between activated and resting Tregs, as well as between activated Tregs and all other cell types represented in these datasets*, CCR8* was found to be the gene that most specifically identified activated Tregs (Fig. 1B). In contrast, other well-known Treg targets such as *CD25 (IL2RA), GITR (TNFSRF18), OX40 (TNFRSF4), CTLA-4,* and *FoxP3* were found to be differentially expressed between Tregs and other immune cells, but only moderately or not upregulated in activated Tregs compared to resting Tregs. The higher specificity of CCR8 compared to CCR4, OX40, GITR, and CD25 for activated Tregs was further confirmed on protein level using flow cytometry on anti-CD3/anti-CD28 stimulated PBMCs (Fig. 1C). Similarly, CCR8 specifically marks the activated Tregs also in mice [41–43]. Thus, specific expression of CCR8 on activated tumor-infiltrating Tregs suggests that therapeutic antibodies against CCR8 may more specifically target these immunosuppressive cells within the TME than previous systemic Treg-targeting approaches.

### Robust antitumor activity of anti-CCR8 antibodies requires Fc-mediated effector function and presence of CD8+ T cells

To preclinically investigate the antitumor potential of CCR8 targeting antibodies, due to modest 70% sequence homology between human and murine CCR8 proteins and lack of cross-reactive antibodies, we first engineered mouse-specific surrogate antibodies (Suppl Table S1). We produced anti-mouse CCR8 antibodies in three different isotypes: chimerized to mIgG2a (mIgG2a), human IgG1 (hIgG1), and in the Fc-silent hIgG1-aglycosylated (N297A) version [50], because they might be different in the mechanisms they mediate and thus might help to dissect the requisite mechanisms for antitumor activity.

Surface Plasmon Resonance (SPR) analysis confirmed the mIgG2a antibody binding to mouse FcγRIII and mouse FcγRIV, crucial for NK cell-mediated ADCC and macrophage-mediated ADCP in mice, respectively (Suppl Table S2) [13, 23, 51]. In contrast, the hIgG1 variant lacked the mouse FcγRIII binding, but strongly bound murine FcγRIV, a receptor that plays a similar role as human FcγRIIA [51]. The Fc-silenced hIgG1-aglycosylated variant showed no binding to mouse FcγRs required for ADCC and ADCP in mice (Suppl Table S2). All three antibodies exhibited selective and potent binding to mouse CCR8 ectopically expressed on HEK293 cells (Suppl Fig S1 A-D), making them suitable tools for investigating the CCR8-specific Treg-targeting in anticancer therapies.

**Fig. 2:**
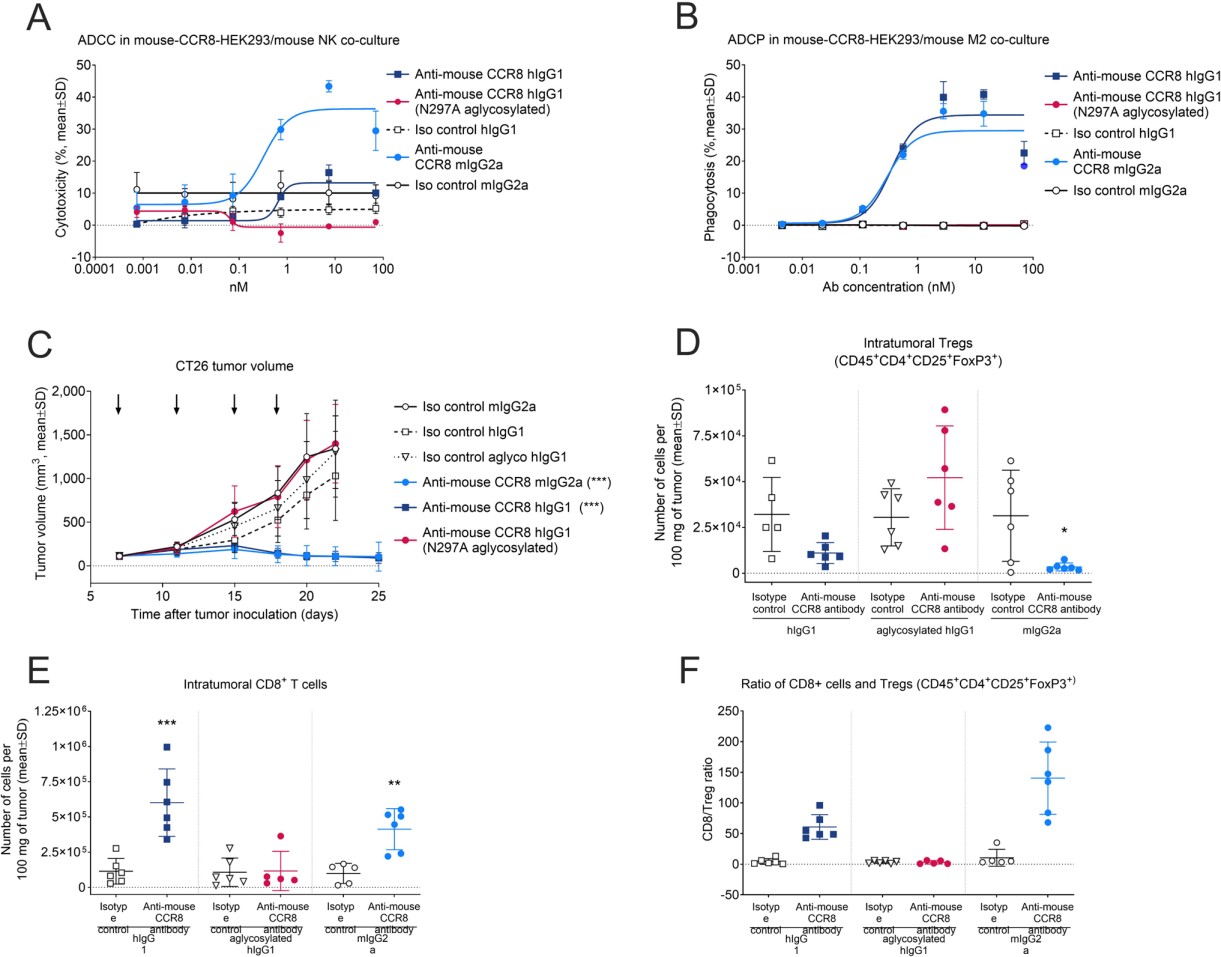
Robust preclinical antitumor activity of anti-mouse CCR8 antibodies is mediated via Fc-mediated effector functions of antibodies inducing ADCC and ADCP. **A** ADCC activity of anti-mouse CCR8 mIgG2a, hIgG1, and N297A-aglycosylated hIgG1 antibody variants and respective non-binding isotype controls, in co-culture of mouse CCR8 expressing HEK293 target cells and primary mouse NK cells as effector cells, at effector/target (E:T) cell ratio of 10:1 (*n* = 2). Cytotoxicity was determined by measuring target cell apoptosis induction relative to the no-antibody control at the 20-h co-culture time point. **B** ADCP activity (real-time) of anti-mouse CCR8 mIgG2a, hIgG1, and N297A-aglycosylated hIgG1 antibody variants and respective non-binding isotype controls, in co-cultures of mouse CCR8 expressing HEK293 target cells and mouse M2 macrophages as effector cells, at effector/target (E:T) cell ratio of 4:1 (*n* = 3). The percentage of phagocytosed target cells was determined by measuring phagocytosis relative to no-antibody control at 4-h co-culture time point. **C** Tumor growth inhibition in mice bearing CT26 tumor and treated with the anti-mouse CCR8 mIgG2a, hIgG1, and N297A-aglycosylated hIgG1 antibody variants, and the respective non-binding isotype controls (all at 10 mg/kg, Q3/4D, i.p., *n* = 10 mice/group). Black arrows indicate antibody treatment days (days 7, 11, 15, and 18) relative to tumor inoculation. Treatment with anti-mouse CCR8 mIgG2a and hIgG1 antibodies resulted in treatment/control (T/C) values of 0.11 (*p* < 0.001) and 0.02 (*p* < 0.001), respectively. T/C: treatment/control ratio calculated from mean tumor volumes at study end. Statistical analysis was performed using an ANOVA model with contrasts. ***p < 0.001 compared with the respective isotype controls. Q3/4D: every third or fourth day (twice weekly), i.p.: intraperitoneally. **D-E** Flow cytometric analysis of **D** intratumoral Tregs (CD45+CD4+CD25+FoxP3+) and **E** CD8+ T cells in CT26 tumor lysates from study described above (**C**). Tumors were collected 24 h after the second treatment dose (*n* = 6 mice/group). Statistical analysis was performed using an ANOVA model with contrasts. *p < 0.05, **p < 0.01, ***p < 0.001 compared with the respective isotype control. **F** Calculated ratio of intratumor CD8 + T cells to Tregs (CD45+CD4+CD25+FoxP3+) as calculated based on data in (D) and (E), 24 h after second antibody treatment (*n* = 6 mice/group)

In vitro, we assessed the ADCC and ADCP activities of the anti-mouse CCR8 antibodies using co-culture assays with mouse CCR8-expressing HEK293 cells as targets. For ADCC assay, primary mouse NK cells, that express only mouse FcγRIII, were used as effector cells. In this setting, anti-mouse CCR8 mIgG2a antibody demonstrated potent cytotoxicity (EC_50_ of 323 pM, with 36% maximal response) outperforming the hIgG1 variant, which showed only residual activity (13% maximal response) that was just above the baseline levels of negative controls (5–10% maximal response) (Fig. 2A). However, in ADCP assay using mouse M2 macrophages, that express both mouse FcγRIII and FcγRIV, both mIgG2a and hIgG1 anti-mouse CCR8 antibodies were similarly effective (EC_50_s of 295 pM and 351 pM, with maximal responses of 29% and 34%, respectively) (Fig. 2B). In both ADCC and ADCP assays, "hook effects" were observed at the highest doses [52], which may have some impact on EC_50_ estimation. Nevertheless, the mIgG2a isotype was highly potent in inducing both ADCC and ADCP, whereas the hIgG1 isotype was only effective in ADCP, and the hIgG1-aglycosylated variant was inactive in both mechanisms, consistent with their respective mouse FcγR binding profiles (Suppl Table S2).

In vivo, using the syngeneic CT26 murine tumor model, monotherapy with the anti-mouse CCR8 hIgG1 antibody achieved strong tumor growth inhibition comparable to that seen with anti-mouse CCR8 mIgG2a treatment (treatment/control (T/C) tumor volume ratios: 0.11 and 0.02, respectively; *p* < 0.001), despite solely mediating ADCP (Fig. 2C). The hIgG1-aglycosylated variant showed no antitumor efficacy although all three antibodies showed comparable CCR8 binding affinity and plasma exposure (Suppl Table S3). Treatment tolerability was confirmed by stable body weight across all groups and absence of immune-related adverse events (Suppl Fig S2A). Tumor growth inhibition was associated with intratumoral Treg reduction (Fig. 2D), and concomitantly increased CD8+ T cell infiltration (Fig. 2E), with the CD8+ T cell/Treg ratio serving as a robust PD biomarker of responses (Fig. 2F). Such immunological changes were not observed with the Fc-silenced hIgG1-aglycosylated antibody variant, consistent with its lack of in vitro activity.

Finally, to determine whether CD8+ T cells are required for the antitumor effects obtained by depleting Tregs, we used transgenic mice expressing diphtheria toxin (DT) receptor under the control of the *CD8A* promoter. Treating such MC38 tumor-bearing mice with DT efficiently removed CD8+ T cells from MC38 tumors and peripheral blood (data not shown) and completely abrogated the in vivo efficacy of anti-CCR8 antibody treatments (Suppl Fig S2B), underscoring the essential role of CD8+ T cells in mediating tumor regression.

These findings suggest that effectiveness of CCR8-targeting antibodies in cancer therapy depends on their ability to engage effector immune cells and remove Tregs, where ADCP might be the key effector mechanism for Treg depletion within the TME.

### The effectiveness of anti-CCR8 antibodies relies on lowering the intratumoral Treg levels below a threshold of 50%

To explore how intratumoral Treg depletion correlates with antitumor responses in vivo, first we treated syngeneic EMT6 tumor-bearing mice with escalating doses of anti-mouse CCR8 mIgG2a antibody (ranging from 0.01 to 10 mg/kg). We observed that tumor growth inhibition correlated with levels of intratumoral Treg reduction and CD8+ T cell infiltration, and that these effects showed steep antibody dose-dependency (Suppl Fig S3A-F). Namely, while treatments with 0.1 mg/kg anti-mouse CCR8 mIgG2a antibody showed no significant effects on tumor size (T/C ratio of 0.9; *p* < 0.5) and immune cells in the TME, the next dose of 1 mg/kg already approached the maximal effect size in terms of in vivo tumor growth inhibition (T/C ratio 0.45; *p* < 0.01), intratumoral Treg reduction and subsequent CD8+ T cell influx.

**Fig. 3:**
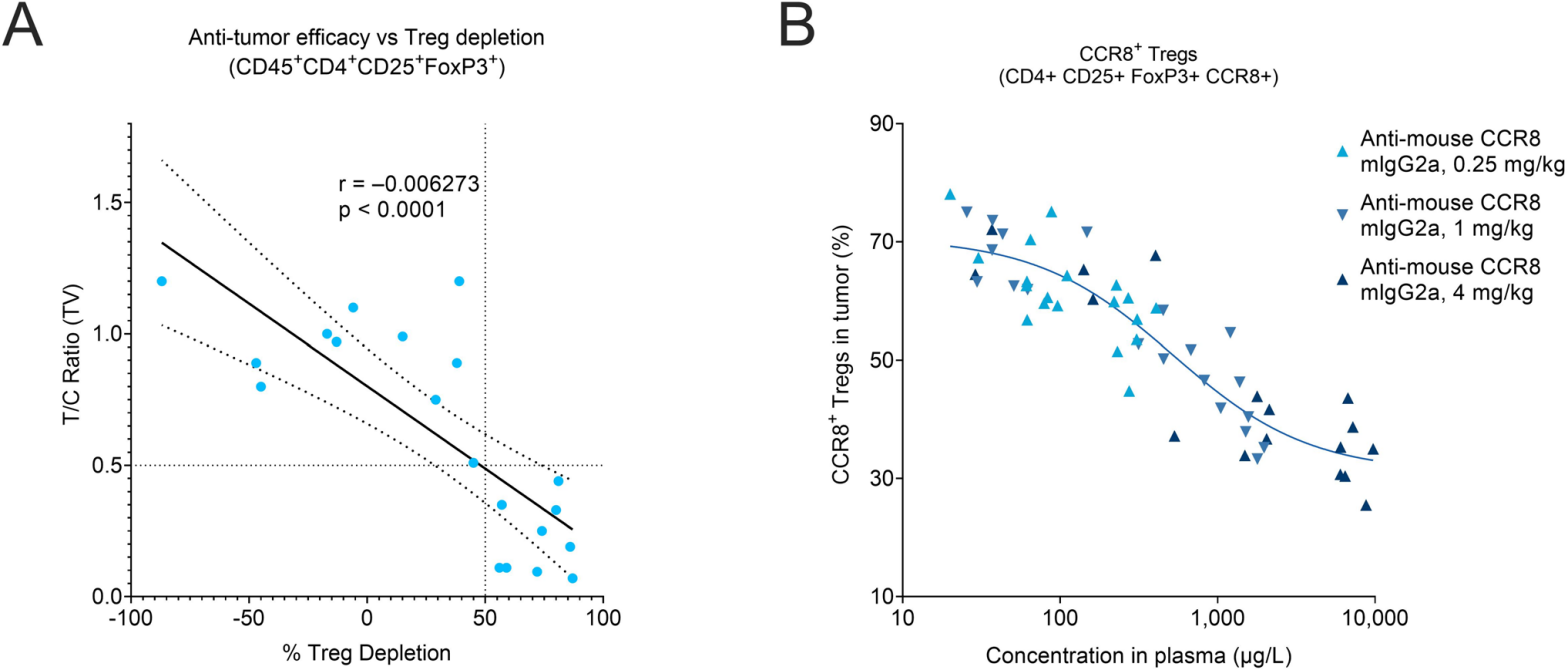
Correlation of the intratumoral Treg depletion efficacy with antitumor responses and plasma exposure of anti-mouse CCR8 antibodies. **A** Correlation between the intratumoral Treg depletion and in vivo antitumor efficacy and anti-mouse CCR8 antibodies (hIgG1 and mIgG2a), across three independent studies using two different mouse tumor models (CT26 and EMT6). Treg depletion efficacy was analyzed by flow cytometry 24 h after second treatment dose and determined in percentages (%) relative to isotype control (*n* = 5–6 mice/group). T/C ratio calculated from mean tumor volumes at study end (*n* = 10 mice/group). The solid and dashed lines indicate the regression line and the associated 95% confidence intervals, respectively. **B** Correlation between the plasma exposure of anti-mouse CCR8 mIgG2a antibody and depletion of intratumoral CCR8 + Tregs (CD4+CD25+FoxP3+CCR8+) in EMT6 tumor-bearing mice treated with a single dose of antibody (0.25, 1, or 4 mg/kg; i.p.), as determined on different time points upon treatment (2, 24, 48, 120, 192 and 336 h, *n* = 5 mice/group/time point)

Next, to better understand to what extent Treg depletion was required for effective tumor growth inhibition in vivo, we evaluated data from three independent in vivo studies employing syngeneic CT26 and EMT6 tumor models treated with varying doses of either mIgG2a or hIgG1 anti-mouse CCR8 antibodies. Our analysis identified a clear empirical correlation: tumor growth was inhibited by more than 50% (i.e., T/C ratio < 0.5) when Treg depletion exceeded 50% from baseline, highlighting a threshold for eliciting significant tumor shrinkage in vivo (Fig. 3A). This could be because not every Treg in the TME express CCR8. Finally, a separate study in the EMT6 model demonstrated that intratumoral CCR8+ Treg depletion, upon a single dose treatment, was directly proportional to the plasma concentration of anti-mouse CCR8 mIgG2a antibody (Fig. 3B; Suppl Fig S4; Suppl Table S4). These data indicate that antitumor activity requires sustained antibody concentrations to maintain Treg levels below 50%.

### Combination of CCR8+ Treg depletion with immune checkpoint blockade enhances antitumor responses

**Fig. 4:**
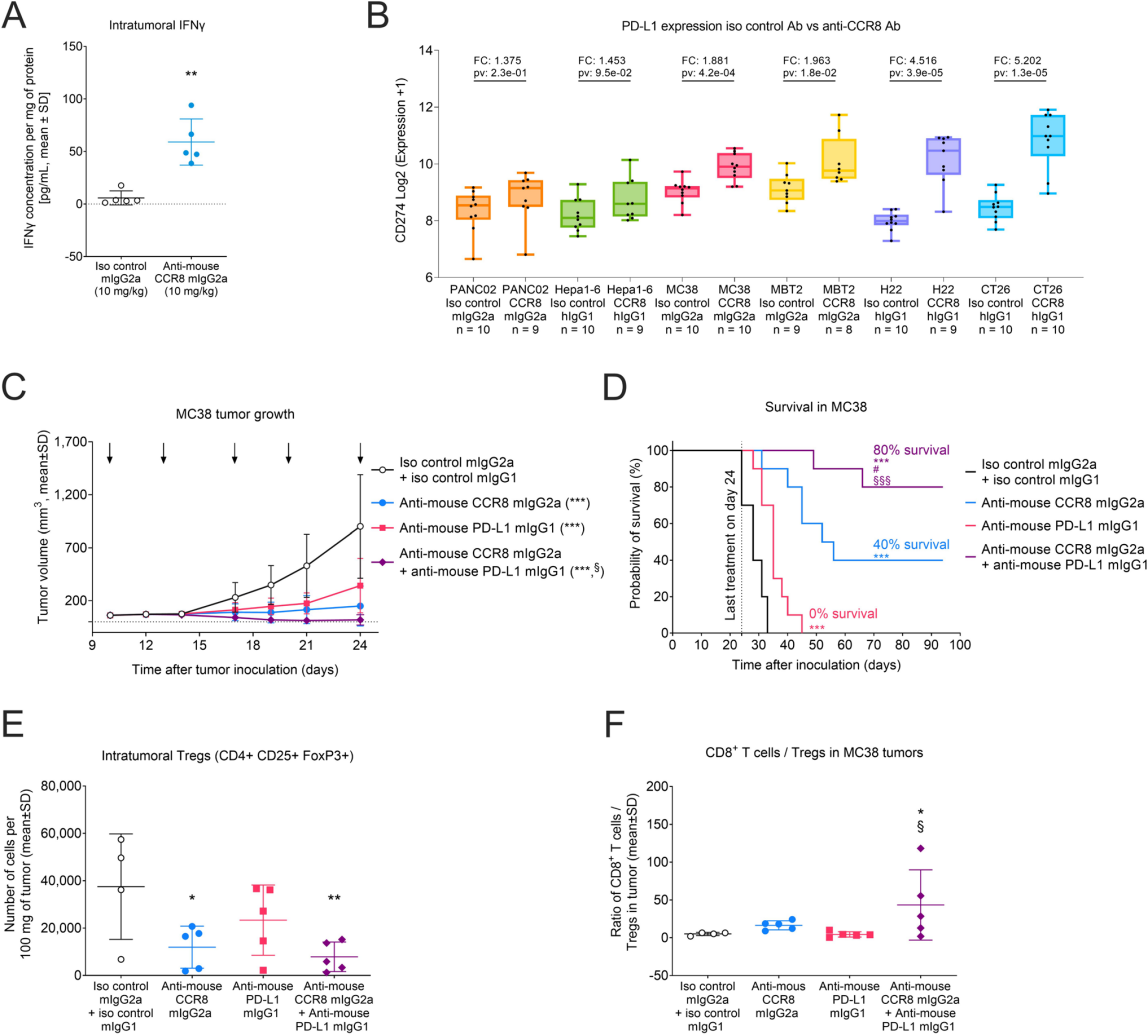
Combination of CCR8 + Treg depletion with anti-PD-L1 immune checkpoint blockade results in enhanced antitumor activity. **A** EMT6 tumor-bearing mice were treated with anti-mouse CCR8 mIgG2a antibody and with the respective non-binding isotype control, and intratumoral IFNγ protein concentration was measured with ELISA on day 19 at study end (*n* = 5). **p < 0.01 indicate statistical significance in comparison to isotype control. **B** Intratumoral *PD-L1* (CD274) expression as determined by RNA-Seq upon treatment of PANC02, Hepa1-6, MBT2, MC38, H22, and CT26 syngeneic tumor-bearing mice with anti-mouse CCR8 mIgG2a and hIgG1 antibody variants and with the respective non-binding isotype controls (*n* = 8–10 mice/group). Log2 fold change expressions were 1.375 in PANC02, 1.453 in Hepa1-6, 1.963 in MBT2, 2.669 in MC38, 4.516 in H22 and 5.202 in CT26 model. **C** Efficacy of anti-mouse CCR8 mIgG2a antibody and anti-PD-L1 antibody as monotherapy and as combination treatment in syngeneic MC38 murine tumor model (also referred as C38). Tumor growth in mice treated with non-binding isotype controls mIgG2a (10 mg/kg, Q3/4Dx5, i.p.) and mIgG1 (3 mg/kg, Q3/4Dx5, i.p.), anti-mouse CCR8 mIgG2a antibody (10 mg/kg, Q3/4Dx5, i.p.), anti-PD-L1 mIgG1 antibody (3 mg/kg, Q3/4Dx5, i.p.), or the combination of anti-mouse CCR8 mIgG2a (10 mg/kg, Q3/4Dx5, i.p.) and anti-PD-L1 mIgG1 antibodies (3 mg/kg, Q3/4Dx5, i.p.). Black arrows indicate treatment days (days 10, 13, 17, 20, and 24) relative to tumor inoculation (*n* = 10 mice/group). Treatment with anti-mouse CCR8 mIgG2a and anti-PD-L1 antibodies resulted in T/C values of 0.17 (*p* < 0.001) and 0.38 (*p* < 0.001), respectively. Combinatorial treatment with anti-mouse CCR8 mIgG2a and anti-PD-L1 antibodies resulted in T/C of 0.02 (*p* < 0.001). Statistical analysis was performed using an ANOVA model with contrasts. ***p < 0.001 is significance in comparison to isotype controls, ^§^*p* < 0.05 in comparison to anti-PD-L1 monotherapy. **D** Survival of mice described in (C). The treatments started on day 10 and the last treatment doses were given on day 24. Mice were sacrificed individually when they met the predefined termination criteria (tumor area of 220 mm^2^). The survival times from inoculation to sacrifice are presented as Kaplan–Meier survival plots. Asterisks, hashtags, and section signs indicate statistical significance in comparison to isotype controls, anti-mouse CCR8 monotherapy, and anti-PD-L1 monotherapy, respectively (^#^*p* < 0.05, ***,^§§§^*p* < 0.001; *n* = 10 mice/group). **E** Intratumoral Tregs (CD4+CD25+FoxP3+) and **F** ratio of CD8 + T cells to Tregs were determined by flow cytometry in MC38 tumors of mice treated as described in (C). Tumors were collected 24 h after the second treatment dose on day 14. Asterisks and section signs indicate statistical significance in comparison to isotype control and anti-PD-L1 monotherapy, respectively (*,^§^*p* < 0.05, **p < 0.01, *n* = 5/group)

Depletion of CCR8+ Tregs had consistent monotherapeutic antitumor activity across several syngeneic murine tumor models (23 models tested), yet the extent of these responses varied and appeared unrelated to the cancer cells’ tissue origin, or the mouse strains used (Suppl Table S5). Consequently, we aimed to further investigate the underlying mechanisms to identify potential candidates for combination drug therapies to improve responses. Therefore, to more broadly assess the changes in the TME associated with anti-CCR8 antibody treatments, we performed RNA-seq on tumor samples from six different syngeneic mouse tumors (PANC02, Hepa1-6, MBT2, MC38, H22, and CT26) treated with either 10 mg/kg anti-mouse CCR8 mIgG2a and hIgG1 antibodies or with respective non-binding isotype controls, and found a notable increase in *CD8* and *IFNγ* mRNA expressions (Suppl Fig S5A-B), associated with anti-CCR8 treatments. An increase in IFNγ protein levels was also confirmed using ELISA (Fig. 4A, Suppl Fig S3G-H), which may explain the subsequent rise in intratumoral expression of the immune checkpoint molecule *PD-L1* (Fig. 4B), known to tone down immune responses [12]. Thus, the observed increase in PD-L1 expression following anti-CCR8 therapy could dampen the immune responses, potentially limiting the effectiveness of the anti-CCR8 therapies. To circumvent this limitation, we combined CCR8+ Treg depletion with anti-PD-L1 blockade in the syngeneic MC38 tumor model (also referred as C38) and observed significantly improved antitumor efficacy (T/C ratio of 0.02) compared to both monotherapies (T/C ratio of 0.17 for anti-CCR8, and 0.38 for anti-PD-L1) (Fig. 4C). While the moderate tumor growth inhibition of anti-PD-L1 monotherapy did not translate into survival benefit by the study end on day 94, anti-mouse CCR8 mIgG2a treatment resulted in a superior survival rate of 40% at study end and led to complete tumor regressions. The combination therapy yielded an improved survival benefit of 80% at study end (Fig. 4D), likely due to further significant elevation of the CD8+ T cell/Treg ratio in the tumors (Fig. 4E-F).

Finally, we also investigated the combination potential of CCR8+ Treg depletion with ICIs in tumor models known to be weak or non-responsive to ICIs. In the MBT2 and MB49 syngeneic tumor models, anti-PD-1 monotherapy elicited minimal responses (T/C ration of 0.66 and 0.63, respectively), while anti-mouse CCR8 mIgG2a antibody treatment produced only moderate responses (T/C ration of 0.5 and 0.37, respectively) (Suppl Fig S5C-D). Importantly, combining these two therapeutic antibodies significantly enhanced the tumor growth inhibition in both MBT2 and MB48 models (T/C ratio of 0.15 and 0.16, respectively), again likely due to further significant elevation of the CD8+ T cell/Treg ratio in the tumors (Suppl Fig S5E-F). Together these data suggest that the full potential of anti-CCR8 immunotherapies may require the concurrent blockade of immune checkpoints with either anti-PD-L1 or anti-PD-1 antibodies, and that tumors that are less responsive to such therapies might be re-sensitized to ICIs by depletion of the immunosuppressive CCR8+ Tregs.

### Depletion of CCR8+ Tregs induces long-term transplantable antitumor immunological memory

**Fig. 5:**
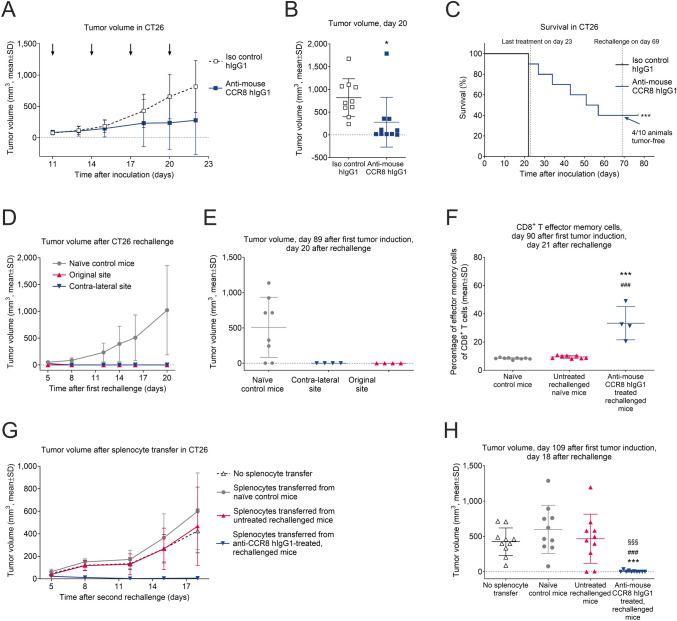
Depletion of CCR8 + Tregs induces long-term transplantable antitumor immunological memory*.* **A** CT26 tumor growth in mice treated with non-binding isotype control (10 mg/kg, Q3Dx4, i.p.) or anti-mouse CCR8 hIgG1 antibody (10 mg/kg, Q3Dx4, i.p.,  *n*= 10 mice/groups). Black arrows indicate treatment days (days 11, 14, 17, and 20). **B** Tumor volumes in mice described in (A) on day 22. Statistical analysis was performed using an ANOVA model with contrasts. *p < 0.05 compared with the isotype control. **C** Survival benefit of anti-mouse CCR8 hIgG1 antibody in mice as described in (A). Last treatment was on day 23. The survival time from inoculation to sacrifice was recorded and reported as Kaplan–Meier survival plots. Asterisks indicate statistical significance in comparison with the isotype control group (***p < 0.001; *n* =10). **D** Tumor growth in mice of the first re-challenge study, where tumor-free (*n* = 4) and age-matched treatment naïve (*n* = 8) mice were challenged by an injection of CT26 cancer cells. In case of the tumor-free mice, re-challenge was performed on the contra-lateral flank 69 days after the original tumor challenge. **E** Tumor volumes in mice described in (D) on day 20 after re-challenge, that corresponds to day 89 after the original tumor challenge. **F** Splenic CD8 + effector memory T cells (CD8+CD44+CD62L-) collected on day 90 from the re-challenged tumor-free mice (*n* = 4), or from untreated age-matched CT26 tumor challenged mice (*n*= 8) described in (D), and from age-matched naïve control mice (*n*= 9). Statistical analysis was performed using an ANOVA model with contrasts. ***p < 0.001 compared with untreated CT26 challenged mice, while ^###^*p* < 0.001 compared with naïve control mice. **G** Tumor growth in mice of the second re-challenge study. Naïve mice (*n* = 10 mice/group) were first injected with splenocytes collected on day 90 from the surviving mice of the first re-challenge study, or with splenocytes collected from untreated but CT26 tumor challenged mice or from naïve mice. One day latter mice were inoculated with CT26 cancer cells, and tumor growth was monitored for another 18 days. **H** Tumor volumes of mice described in (G) on day 18 after tumor inoculation. Statistical analysis was performed using an ANOVA model with contrasts. ^§§§^*p* < 0.001 compared with the no splenocyte transfer group, ***p < 0.001 compared with naïve control mice’s splenocyte transfer; ^###^*p* < 0.001 compared with splenocyte transfer of untreated mice that were challenged with CT26

Next, we sought to determine whether depletion of the immunosuppressive CCR8+ Tregs from the TME could trigger long-lasting antitumor immunological memory, crucial for persistent tumor immune surveillance and durable responses to cancer therapy. First, we treated CT26 tumor-bearing mice with 10 mg/kg of anti-mouse CCR8 hIgG1 antibody until day 20, which strongly suppressed tumor growth (Fig. 5A-B) and led to complete tumor elimination in 40% of mice by day 69 post-inoculation (Fig. 5C). These tumor-free surviving mice were then re-challenged with CT26 cancer cells on the contra-lateral side and both the original and new sides were monitored for 20 more days without further treatments. Unlike the age-matched un-treated control mice, the re-challenged mice showed no tumor growth on either side, indicating they were fully protected against CT26 cancer cells (Fig. 5D-E). Additionally, we found a marked increase in CD8+ T effector memory cells (CD8+ CD44+ CD62L-) in the spleens of mice that had been pretreated with anti-CCR8 antibody and then re-challenged, compared to untreated or naïve control mice (Fig. 5F). To conclusively demonstrate lasting immune memory and protection against tumor recurrence, we finally transferred splenocytes from tumor-free pretreated and re-challenged mice (collected on day 90), along with splenocytes from naïve mice, and from untreated tumor challenged mice, into new recipients. These mice were then challenged with CT26 cancer cells one day later, and tumor growth was tracked for 18 days. Only the splenocytes from mice that had been pretreated with the anti-CCR8 antibody and re-challenged prevented tumor growth (Fig. 5G-H). These findings suggest that anti-CCR8 therapies may allow for development of long-lasting antitumor immune memory leading to durable responses. Collectively, our preclinical data prompted us to pursue the development of anti-human CCR8 antibodies for the treatment of human cancers.

### Fc-optimized anti-human CCR8 antibody BAY 3375968 mediates potent ADCC and ADCP of cells expressing human CCR8

To specifically target and eliminate CCR8+ Tregs in human tumors, we engineered two anti-human CCR8 hIgG1 antibodies: BAY 3353497 with conventional glycosylation, and BAY 3375968 as an afucosylated variant. We selected the hIgG1 isotype for its high affinity to human FcγRs, which are crucial for eliciting ADCC and ADCP [13, 14]. Additionally, we enhanced the interaction with human FcγRIIIA through Fc engineering (afucosylation) to boost ADCC [15], a modification that could be particularly advantageous in TMEs with low NK cell infiltration. SPR analysis confirmed that afucosylation of BAY 3375968 significantly increased its binding affinity to human FcγRIIIA variants by around tenfold over its conventionally glycosylated counterpart, BAY 3353497, with similarly enhanced binding strengths to both, the high- (V158) and low-affinity (F158) variants (Suppl Table S6). However, both antibodies showed potent and comparable binding to the high-affinity FcγRI and to FcRn, and they showed no measurable binding to the inhibitory FcγRIIB (Suppl Table S6). To create a fully Fc-silenced control antibody that would eliminate all FcγR interactions necessary for ADCC and ADCP, we generated a LALA-aglycosylated antibody variant (L234A, L235A, N297A) [53]. All three antibodies displayed equipotent cellular binding affinity to the human CCR8 ectopically expressed on HEK293 cells (data not shown) and CHO cells (Suppl Fig S6A-B). These data position BAY 3375968 as a suitable candidate for further preclinical studies in targeting human CCR8 positive Tregs.

**Fig. 6:**
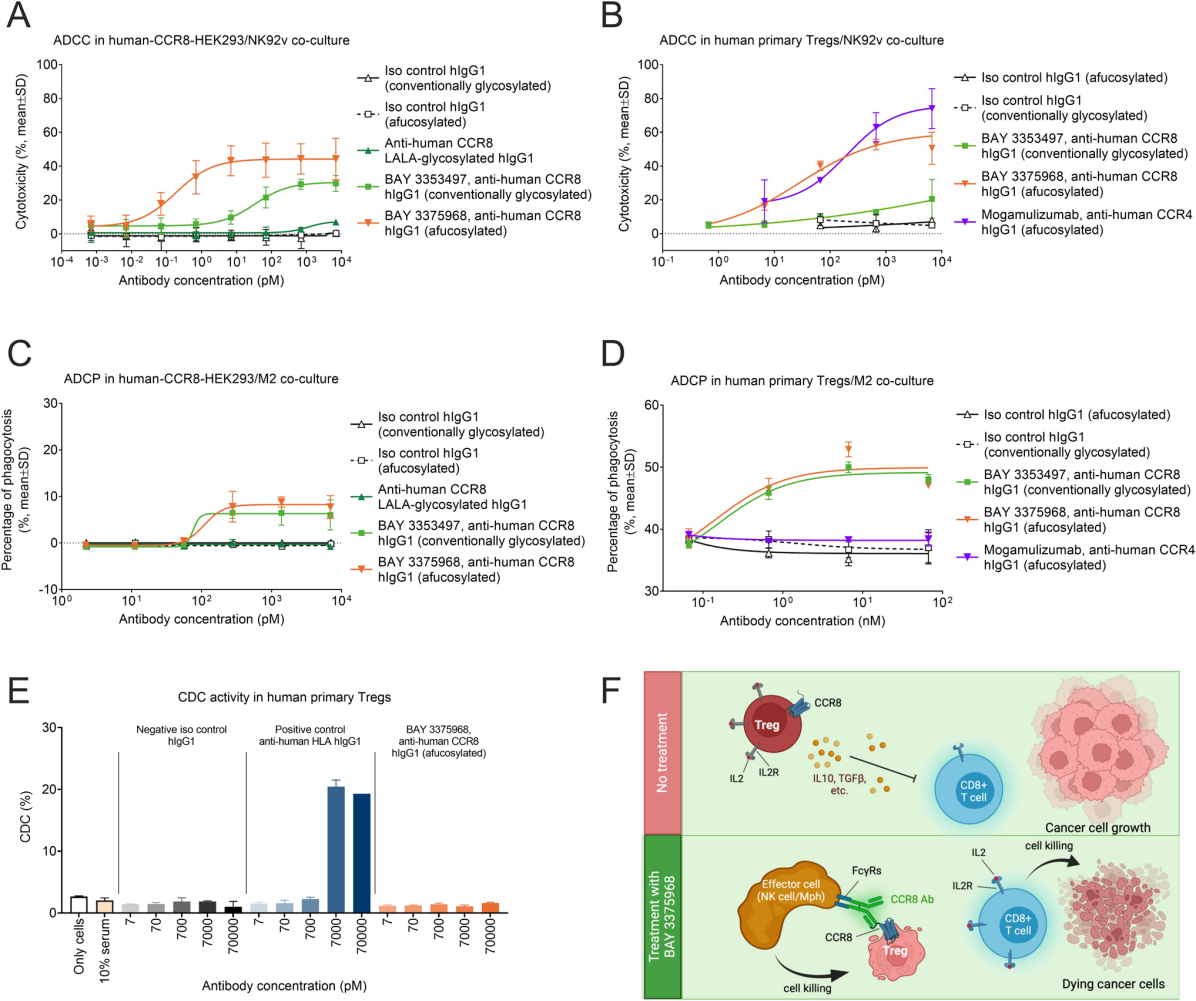
The afucosylated anti-human CCR8 antibody BAY 3375968 induces potent ADCC and ADCP but does not stimulate CDC. **A** ADCC activity of afucosylated anti-human CCR8 hIgG1 antibody BAY 3375968, conventionally glycosylated anti-human CCR8 hIgG1 antibody BAY 3353497, LALA-aglycosylated anti-human CCR8 hIgG1 and afucosylated or conventionally glycosylated hIgG1 non-binding isotype controls, in co-culture of human CCR8-expressing HEK293 target cells and human NK92v effector cells, at an E:T ratio of 4:1 (*n* = 3). Cytotoxicity was determined by measuring target cell apoptosis induction relative to the no-antibody control at the 4-h co-culture time point. **B** ADCC activity of anti-human CCR8 antibodies BAY 3375968 and BAY 3353497 in comparison of afucosylated anti-human CCR4 hIgG1 antibody mogamulizumab and the respective non-binding isotype controls, in co-culture of primary human activated Tregs as target cells and human NK92v effector cells, at an E:T ratio of 4:1 (*n* = 3). Cytotoxicity was determined by measuring the change in target cell quantity relative to a no-antibody control at the 2-h co-culture time point. Data are from one representative Treg donor with 85% CCR8 expression with technical replicates. **C** ADCP activity (real-time) of afucosylated anti-human CCR8 hIgG1 antibody BAY 3375968, conventionally glycosylated anti-human CCR8 hIgG1 antibody BAY 3353497, LALA-aglycosylated anti-human CCR8 hIgG1 and afucosylated or conventionally glycosylated hIgG1 non-binding isotype controls, in co-culture of human CCR8-expressing HEK293 target cells and primary human M2 macrophages as effector cells, at E:T ratio of 10:1 (*n* = 3). Percentage of phagocytosed target cells was determined by measuring phagocytosis relative to no-antibody control at the 24-h co-culture time point. **D** ADCP activity (real-time) of anti-human CCR8 antibodies BAY 3375968 and BAY 3353497 in comparison of afucosylated anti-human CCR4 hIgG1 antibody mogamulizumab and the respective non-binding isotype controls, in co-culture of primary human Tregs as target cells and primary human M2 macrophages as effector cells, at an E:T ratio of 4:1 (*n* = 3). Here phagocytosis was determined as percentage of effector cells phagocyting (CFSE + CD206 + double-positive effector cells) at the 4-h co-culture time point. Data are from one representative Treg donor with 63% CCR8 expression with technical replicates. **E** CDC activity of afucosylated anti-human CCR8 hIgG1 antibody BAY 3375968, a positive control anti-human HLA-I hIgG1 antibody, and a negative non-binding isotype control hIgG1 antibody. Primary human Tregs with more than 80% CCR8 expression (*n* = 2 donors) were exposed to CDC-qualified human complement serum and the above antibodies in dose–response manner. Cell death caused by CDC was quantified using flow cytometry following 7-AAD staining. A representative donor is shown with technical replicates. **F** Mode-of-action: activated CCR8 + Tregs, primarily residing in TME, suppress the antitumor immune function of cytotoxic T cells via various mechanisms including the sequestration of IL-2 and release of immunosuppressive cytokines such as IL-10 and TGFβ. BAY 3375968 engages effector cells and induces targeted depletion of CCR8 + Tregs, leading to enhanced CD8 + T cell activation and subsequent tumor cell destruction

Next, we evaluated the cellular mechanisms-of-action of BAY 3375968 through co-culture assays using various human CCR8-expressing target cells. In vitro ADCC activity was first assessed using human CCR8-expressing HEK293 cells as targets and NK92v cells as effector cells. BAY 3375968 demonstrated significantly stronger ADCC activity, and a higher maximal response compared to BAY 3353497 (EC_50_: 0.35 pM and 34.1 pM with maximal responses of 40.9% and 28.3%, respectively) (Fig. 6A; Suppl Table S7). The Fc-silenced LALA-aglycosylated variant of the BAY 3375968 antibody showed no ADCC activity, confirming that cytotoxicity was Fc-dependent and that the enhanced human FcγRIIIA engagement, due to afucosylation, was responsible for the increased ADCC potency of BAY 3375968. This finding was further supported by ADCC assay using primary human NK cells, where BAY 3375968 outperformed its conventionally glycosylated counterpart (EC_50_: 0.48 pM and 8.1 pM with maximal responses of 32.7% and 26.3%, respectively), showing consistency across different experimental setups (Suppl Fig S7A; Suppl Table S7). Moreover, BAY 3375968 also exhibited superior ADCC activity compared to BAY 3353497 in assays using primary human activated Tregs as target cells, while its effectiveness was comparable to the FDA-approved mogamulizumab, a CCR4 depleting afucosylated hIgG1 antibody (Fig. 6B) [36, 37].

For in vitro ADCP activity, which was sufficient by itself for effectively depleting CCR8+ Tregs from mouse tumors in vivo, we used a co-culture assay with human CCR8-expressing HEK293 cells as targets and primary human M2 macrophages as effector cells. Interestingly, BAY 3375968 and BAY 3353497 had similar potency (EC_50_: 108 pM and 76 pM with maximal responses of 8.3% and 6.3%, respectively), while the Fc-silenced antibody variant was inactive (Fig. 6C), indicating that induction of phagocytosis was also Fc-dependent, and that afucosylation, at least under these experimental conditions, did not enhance ADCP. This is in alignment with the fact that afucosylation does not increase the affinity of BAY 3375968 to human FcγRIIA (Suppl Table S6), the primary receptor mediating ADCP in humans [17, 23]. Induction of phagocytosis was highly efficient, and 68% of target cells were phagocytosed within the first 24 h (Suppl Fig S7B). In ADCP assays with primary human activated Tregs as target cells, BAY 3375968 showed also comparable activity to BAY 3353497, but compared favorably to mogamulizumab, which exhibited no ADCP activity in our assay conditions (Fig. 6D). This puzzling ADCP activity difference between our anti-CCR8 antibodies and mogamulizumab may be attributed to the higher CCR8 expression on human activated Tregs compared to CCR4.

Finally, BAY 3375968 was also assessed for in vitro complement-dependent cytotoxicity (CDC) using primary human Tregs. While a positive control antibody anti-human HLA complex I hIgG1 induced strong CDC, BAY 3375968 did not (Fig. 6E).

In conclusion, BAY 3375968 has demonstrated the ability to effectively deplete CCR8+ human Tregs via ADCC and ADCP mechanisms in vitro, with its ADCP activity showing an advantage over mogamulizumab. The preclinical evidence of BAY 3375968's mode-of-action in targeting CCR8 (Fig. 6F) supports its clinical development as anticancer therapy.

## Discussion

Tregs are immunosuppressive cells that play an indispensable role in maintaining peripheral tolerance to prevent undesired autoimmune reactions. Thus, deficiency in Treg numbers or functionality can result in various autoimmune diseases, while a high presence of Tregs and a high Treg to CD8+ T effector cell ratio can suppress cancer immune surveillance and contribute to cancer progression [3, 6, 8, 9]. Along these lines, Tregs have also been identified as one of the major mechanism of resistances to ICI therapies [1, 2, 54–56]. Although the idea of depleting Tregs has long been considered for anticancer therapeutic intervention, past attempts have been limited by insufficient selectivity of the drug targets for tumor-residing Tregs, possibly explaining the suboptimal responses and severe immune-related adverse events [31, 57–59]. Here we confirmed CCR8 as a cell surface receptor selectively expressed on activated tumor-residing Tregs, presenting an ideal target to potentially improve therapeutic anticancer responses and reduce the safety concerns associated with the previous systemic Treg-targeting approaches.

Chemokine receptors belong to G-protein coupled receptors that are activated by chemokines whose gradient is crucial for selective trafficking and homing of immune cells, with CCR8-CCL1 axis potentially guiding Tregs to their target sites. As such, some therapeutic approaches have attempted to inhibit CCR8 signaling to block Treg migration into tumors [60]. Yet, other studies [43, 61] suggest that simply binding to and inhibiting CCR8 signaling does not translate into meaningful preclinical antitumor efficacy. This could be attributed to potential functional redundancy among chemokine receptors, suggesting that blocking CCR8 alone may be insufficient if Tregs can use other receptors like CCR4 for migration. Alternatively, the high frequency of activated CCR8+ Tregs within the TME might be due to their local expansion in response to tumor antigens rather than due to trafficking from peripheral tissues, again suggesting that inhibition of migration might not be an effective approach for lowering the number of Tregs within the TME. Finally, our findings here also confirmed a clear necessity for the Fc effector function of anti-CCR8 antibodies for achieving effective inhibition of tumor growth in vivo.

In preclinical models we demonstrated that efficient tumor growth inhibition requires at least 50% reduction of activated Tregs in the TME and a concomitant increase in the CD8+ T cell to Treg ratio, as antitumor efficacy was lost when CD8+ T cells were co-depleted with Tregs. This led us to perform mechanism-of-action studies to better understand how our anti-CCR8 antibodies deplete Tregs within the TME, to ensure that our therapeutic antibodies effectively engage those mechanisms. Our data show that an antibody that only induces ADCP is just as effective in murine tumor models as one that triggers both ADCP and ADCC in removing CCR8+ Tregs and reducing tumor size in vivo. This suggests that ADCP is a primary mechanism of how CCR8+ Tregs are eliminated from tumors and is consistent with findings from other Treg-targeting approaches, such as anti-CTLA-4 antibodies, where it has been shown in mice that Treg depletion rather than functional blockade was necessary, with ADCP being the key effector mechanism [27, 28]. It is notable that like in mouse tumors, human tumors also typically have a higher prevalence of macrophages, the primary mediators of ADCP, than NK cells, which mediate ADCC. However, the dominant mechanism by which depleting antibodies may eliminate CCR8+ Tregs in human tumors are still not fully understood. To ensure the success of our therapeutic antibody, we optimized the Fc part of the antibody to effectively engage both ADCC and ADCP mechanisms in human tumors. We afucosylated the Fc part of the antibody to enhance its binding affinity to human FcγRIIIA and consequently enhanced the ADCC potency of our anti-human CCR8 antibody, BAY 3375968. Interestingly, in contrast to ADCC, afucosylation did not further increase the already strong ADCP activity of the antibody when using blood monocyte-derived M2 macrophage. This might be due to in vitro generated macrophages expressing less human FcγRIIIA than their natural tissue-resident counterparts also found in human tumors [62]. Despite trying various in vitro differentiation assay, we could not identify a protocol that would allow us to significantly enhance the human FcγRIIIA expression on these effector cells, indicating that in vitro assays may not show the full ADCP potential of BAY 3375968 (data not shown). However, the possibility cannot be excluded that human FcγRIIIA plays no major role in mediating ADCP, and that human FcγRIIA might be one the most important activating receptor, as suggested by others [13, 23, 63].

The primary aim of eliminating the suppressive Tregs from tumors is to lift the inhibition on tumor antigen-specific CD8+ T cells, thereby enabling their antitumor activity. In line with this, depletion of CCR8+ Tregs leads not only to a rise in the number and activation of CD8+ T cells but also to elevated IFNγ secretion within the TME. Although important for initial antitumor responses, this increase in CD8+ T cell activity in turn induces the expression of immune checkpoint ligands such as PD-L1, which can function to locally dampen immune responses [12]. Consequently, while preclinical evidence shows that anti-CCR8 therapies are highly effective in monotherapy, achieving durable responses may require combination therapies with immune checkpoint blockade, as confirmed in our preclinical studies where only co-treatment of anti-CCR8 antibodies with ICIs resulted in durable antitumor activity across various mouse models.

Based on our encouraging preclinical data, a Phase I First in Human clinical trial of BAY 3375968 as monotherapy and in combination with ICI pembrolizumab is underway in patients with solid tumors (NCT05537740).

## Supplementary Information

Below is the link to the electronic supplementary material.

Supplementary file1 (DOCX 77 KB)

Supplementary file2 (JPG 775 KB)

Supplementary file3 (JPG 496 KB)

Supplementary file4 (JPG 1518 KB)

Supplementary file5 (JPG 240 KB)

Supplementary file6 (JPG 1824 KB)

Supplementary file7 (JPG 621 KB)

Supplementary file8 (JPG 498 KB)

Supplementary file9 (DOCX 315 KB)

## Data Availability

Data are available from the corresponding author upon reasonable request and with permission of Bayer AG.
