# Supplementary material for: Selective depletion of tumor-infiltrating regulatory T cells with BAY 3375968, a novel Fc-optimized anti-CCR8 antibody
Source: Clin Exp Med. 2024 Jun 10;24(1):122. doi: 10.1007/s10238-024-01362-8 (PMC11164760; doi:10.1007/s10238-024-01362-8)
Supplement: Supplementary file 1 — Supplementary file1 (DOCX 77 KB) [file 10238_2024_1362_MOESM1_ESM.docx]

**Selective depletion of tumor-infiltrating regulatory T cells with BAY 3375968, a novel Fc-optimized anti-CCR8 antibody**

Helge G Roider^1,#^, Sabine Hoff^1,#^, Su-Yi Tseng^1,#^, Sandra Berndt^1^, Mark Trautwein^1^, Katharina Filarsky^1,2^, Uwe Gritzan^1,3^, Jordi Camps^1^, Wiebke Maria Nadler^1^, Joanna Grudzinska-Goebel^1^, Philipp Ellinger^1^, Theresa Pesch^1^, Chai Fen Soon^1^, Marcel Geyer^1^, Katja Gluske^1^, Beatrix Stelte-Ludwig^1,4^, and Mátyás Gorjánácz^1,^*

^1^Bayer AG, Pharmaceuticals

^2^Current address: Roche Diagnostics GmbH, Penzberg, Germany

^3^Current address: Memorial Sloan Kettering Cancer Center, New York, USA

^4^Current address: Vincerx Pharma, Monheim am Rhein, Germany

^#^These authors are co-first authors

*Correspondence to: matyas.gorjanacz@bayer.com

**SUPPLEMENTARY MATERIALS AND METHODS**

**Generation of anti-CCR8 antibodies**

For the generation of antibodies against mouse CCR8, the selection strategy was designed to identify antibodies that bind to the mouse CCR8 tyrosine rich domain (TRD) within the N-terminal region (MDYTMEPNVTMTDYYPDFFTAP). For positive selection on the mouse CCR8 TRD peptides, a mixture containing 50% normal peptide and 50% sulfated peptide was used, since tyrosine sulfation of CCR8 is expected to affect the ligand binding. Four panning rounds were conducted with decreasing concentrations of peptide (1.5, 0.8, 0.4, and 0.2 µM) to increase selection pressure for high-affinity binders. Unwanted binders were removed by a depletion step using a mixture of sulfated and non-sulfated off-target peptide (biotinylated human CCR4 TRD within the N-terminal region; MNPTDIADTTLDESIYSNYYLYESIPKP).

For the generation of antibodies against human CCR8, two selection strategies were designed to identify antibodies that bind to the human CCR8 N-terminal region and cynomolgus CCR8 N-terminal region, respectively. All peptides used were sulfated.

Strategy I was to conduct the first panning round on the human CCR8 N-terminal (MDYTLDLSVTTVTDYYYPDIFSSPSDAELIQTNGK) followed by either three rounds on the same peptide or the cynomolgus CCR8 N-terminal (MDYTLDPSMTTMTDYYYPDSLSSPSDGELIQRNDK). Strategy II was to start with one panning round on the cynomolgus CCR8 N-terminal, followed by either three rounds on the same peptide or the human CCR8 N-terminal. The four panning rounds were conducted with decreasing concentrations of peptide (1.5, 0.75, 0.3, and 0.15 µM or 1.5, 1.5, 0.75, and 0.3 µM, for strategy I and II, respectively) to increase selection pressure for high-affinity binders. Unwanted binders were removed as described for the generation of antibodies against mouse CCR8.

The panning protocol was as follows; a fully human antibody phage display library (BioInvent n-CoDeR Fab lambda library) was used to isolate human monoclonal antibodies by selection against soluble biotinylated peptides provided by Pepscan. For the panning procedures, streptavidin-coupled Dynabeads M-280 (Invitrogen™) were pre-blocked with 300 µL of 3 % milk powder solution in phosphate-buffered saline + 0.05 % Tween 20 (PBST) on an end-over-end mixer at room temperature (RT) for 1 hour. The beads were washed (3x with PBST) and subsequently coated with the biotinylated target or off-target peptide at RT for 1 hour. Dynabeads were washed and subsequently blocked on an end-over-end mixer at RT for 1 hour. For depletion of off-target binders, the blocked phage library was added to the blocked off-target loaded Dynabeads and incubated on an end-over-end mixer at RT for 10 min. This depletion step was repeated twice. The depleted phage library was added to the blocked target loaded Dynabeads and incubated on an end-over-end mixer at RT for 60 min. After stringent washing (3 x in blocking buffer and 9 x in PBST), Dynabeads to which Fab phages bound specifically to the coated target were used directly to infect *Escherichia coli* strain HB101. The phages were subsequently amplified in *Escherichia coli* strain HB101 using the M13KO7 Helper Phage (Invitrogen™).

For each clone pool, 88 randomly picked Fab phage clones were subjected to monoclonal cultivation and expression and subsequently tested for binding to the corresponding target used before to panning. Separate ELISA measurements were conducted for the sulfated and the non-sulfated peptides.

For ELISA screening, peptides were coated onto streptavidin-coated plates (Greiner bio-one, #781997) at a concentration of 0.1 µg/mL in coating buffer (Carbonat-Basis, Candor, #121125) at 4 °C. After the plates were washed 3 times with 60 µL PBS 0.05 % Tween and blocked with 50 µL Smart Block® (Candor, #113500) at 20 °C for 1 hour, 10 µL sFab samples were added to the plates and incubated at 20 °C for 1 hour. After subsequent wash with 60 µL PBS 0.05% Tween 3 times, 20 µL anti-c-Myc HRP antibody (Bethyl Laboratories Inc., #A190-105P, 1:10,000 in PBS 0.05 % Tween 10 % Smart Block) was added and incubated at 20 °C for 1 hour, followed by subsequent wash with 60 µL PBS 0.05 % Tween 3 times, and addition of 20 µL Amplex Red solution (1:1,000 in NaP-buffer 50 mM pH7.6 with 1:10,000 of 30% H_2_O_2_; Invitrogen, #A12222). After a final incubation at 20 °C for 20 min, the signal was determined using an emission wavelength of 595 nm and an excitation of 530 nm. The signal-to-background ratio of individual sFab clones was used for analysis, while background was determined by the value of 8 wells in each plate without sFab culture but with the corresponding sample medium.

In addition, binding of antibodies to cells expressing human, mouse, and cynomolgus CCR8 was determined by flow cytometry.

BAY 3375968 was afucosylated using ProBioGen’s GlymaxX technology where fucose synthesis is redirected by a heterologous enzyme that depletes the fucose pool inside the cell, resulting in the production of afucosylated antibodies at N-glycan on N297.

**Affinity determination of anti-CCR8 antibodies to mouse or human Fragment crystallizable gamma Receptors (FcγRs) using Surface Plasmon Resonance (SPR)**

SPR binding assays were performed on a T200 instrument (Cytiva) at 25 °C with a CM5 sensor chip (Cytiva) and assay buffer HBS-EP+ (Cytiva). Following recombinant mouse FcγR variants were used: FcγRI/CD64 (CD4-M5227, ACROBiosystems), FcγRIIB/CD32b (CDB-M52H7, ACROBiosystems), FcγRIII/CD16 (CDA-M52H8, ACROBiosystems), FcγRIV/CD16-2 (FC4-M52H3, ACROBiosystems). Following recombinant human FcγR variants were used: FcγRI/CD64 (1257-FC, R&D Systems), FcγRIIA/CD32a, isoform R167 (1330-CD/CF, R&D Systems), FcγRIIB/C/CD32b/c (1875-CD, R&D Systems), FcγRIIIA/CD16a, isoform V176 (4325-FC, R&D Systems), FcγRIIIA/CD16a, isoform F176 (CDA-H5220, ACROBiosystems), FcγRIIIB/CD16b (1597-FC, R&D Systems). FcγR (Acro Biosystems) variants were captured via an amine coupled anti-His tag capture antibody (Cytiva) to ~ 25 RU and antibodies were used as analyte at concentrations ranging from 1.5 to 25,000 nM with a flow rate of 30 µl/min. *K*_D_ values were derived from a steady state affinity analysis or from kinetic data fitted to a 1:1 Langmuir isotherm.

**Affinity determination of anti-CCR8 antibodies to mouse or human neonatal Fragment crystallizable Receptor (FcRn) using Surface Plasmon Resonance (SPR)**

To assess the binding affinity of anti-CCR8 antibodies to FcRn, the binding was investigated using SPR at pH 6.0 and pH 7.4. Binding assays were performed on a Biacore T200 instrument (Cytiva) at 25 °C with a CM5 sensor chip (Cytiva) and assay buffer PBS+ (Cytiva) supplemented with 0.05 % surfactant P20 (Cytiva). For FcRn binding assays, recombinant mouse FcRn (FCM-M52W2, ACROBiosystems) and recombinant human FcRn (FCM-H5286, ACROBiosystems) were amine-coupled to the CM5 sensor chip surface (~ 300 RU) and antibodies injected at concentrations ranging from 1.5 – 2,000 nM in PBS+, pH 6 nM, with a flow rate of 30 µl/min. Regeneration was performed with PBS+ pH 7.4. In another experiment at pH 7.4 only, IgGs were tested with one concentration of 2 µM, thus it was only qualitatively assessed if a binding occurs. *K*_D_ values were derived from a steady state affinity analysis by fitting to a 1:1 binding isotherm.

***Ex vivo* analysis of CCR8 protein expression on human peripheral blood mononuclear cells (PBMCs)**

In primary human cells the expression of CCR8 was compared to OX40, CD25, CCR4 and GITR by flow cytometry using frozen human PBMC and CD4+ CD25+ sorted human PBMC cell populations from healthy human donors. PBMCs and sorted CD4+ CD25+ cells were thawed and cultured in TexMACS medium, containing 500 U/ml rIL-2 (R&D Systems) at a cell density of 1 x 10^6^ cells/ml and stimulated by addition of CD3/CD28 MACSiBead particles (130-091-441, T Cell Activation/Expansion Kit, Miltenyi Biotech). After one day, fresh TexMACS medium containing 500 U/ml rIL-2 was added to the cells, followed by a splitting, and feeding step (1:1) at day 3. After another 3 days of incubation at 37 °C and 5 % CO2 in a humidity incubator, target expression was measured by flow cytometry, whereby Fc blocking reagent was used to ensure that only antigen specific binding was observed, and dead cells were excluded in flow cytometry by using Viable dye-APC-Cy7 (65-0865-14, eBiosciences). Following antibodies were used: CD3-V500 (BD Biosciences), CD4-V450 (BD Biosciences), CD8-Pre-Cy5.5 (BD Biosciences), FoxP3-APC (Thermo Fischer Scientific), CD127-PE-Cy7 (BD Biosciences), hCCR8-PE (Biolegend, 360604 L263G8), OX40-BV510 (BD Biosciences), CD25-FITC (Biolegend, 356106 M-A251), CCR4-BV510 (BD Biosciences), GITR-PerCP-eFluor710 (Thermo Fischer Scientific).

**Cell culture and generation of CCR8-expressing CHO and HEK293 cells**

CHO (VGT) cells were cultured in DMEM/Ham's F-12 (1:1), supplemented with 10% fetal bovine serum (FBS), GlutaMAX™-I (Invitrogen, #31331-093), and 0.5 mg/mL G418 (Thermo Fisher Scientific, #10131035**)**. CHO cells were detached with Accutase™ cell detachment solution (Innovative Cell Technologies Inc.) and transfected either with human *CCR8* plasmids, or empty vector (InSCREENex GmbH).

HEK293 (human embryonic kidney 293) cells were cultured in RPMI1640, GlutaMAX Supplement medium (Gibco, #61870-010) supplemented with 10 % fetal calf serum (FCS) (Sigma, #S0615), 1 % Penicillin-Streptomycin (Gibco, #15140-122), and 0.5 mg/mL G418 (Alfa Aesar, #J63871). The cells were maintained at 37 °C in a humidified 5 % CO_2_ incubator. The adherent cells were harvested non-enzymatically (Cell Dissociation Buffer PBS-based, Gibco, #13151-014) and transfected either with mouse *CCR8* plasmids, human *CCR8* plasmid or empty vector (InSCREENex GmbH).

To prepare the transfection mix, plasmid DNA (20 μg/mL) and 293fectin™ Transfection Reagent (1:12.5 dilution, Invitrogen, #12347-019) were diluted in 30 μL Opti-MEM® I Reduced-Serum Medium, (Gibco, #11058-021) and incubated at room temperature for 20 min. Subsequently, 2 x 10^6^ HEK293 cells in 1 mL Freestyle™ F17 Expression Medium (Gibco, #A13835‑01) were plated onto sterile 24-well block plates (QIAGEN, #19583). After 20 min, 50 μL of transfection mix was added to the cells. The plates were shaken at 37 °C, 6 % CO_2_, 400–500 rpm for 4 hours. After adding 1 mL of F17 medium supplemented with 2x penicillin/streptomycin solution, and 0.5 % Gelatine peptone N3 (OrganoTechnie, #19554), the plates were shaken in the same conditions for 24–48 hours.

**Cellular binding of anti-mouse and anti-human CCR8 antibodies to human and murine CCR8 *in vitro***

The *in vitro* binding of the anti-mouse CCR8 antibodies (hIgG1, N297A-aglycosylated hIgG1, or chimerized to mIgG2a) and the respective non-binding isotype control antibodies to mouse CCR8 was studied using mouse CCR8-transfected HEK293 cells, and HEK293 cells transfected with empty vector.

Adherent cells were harvested non-enzymatically (Cell Dissociation Buffer, enzyme-free, PBS; Gibco, #13151-014) and the cells (100,000 cells/well) were suspended in 100 μL FACS Buffer (auto MACS Rinsing Solution, Miltenyi #130-091-222) + 1:20 MACS BSA Stock Solution , Miltenyi #130-091-376)), and stained with the primary antibodies (0.128, 0.64, 3.2, 16, 80, 400, 2000, 10000, 50000, 250000 ng/ml) in 96-well V-Bottom microtiter plates (Falcon, #353263). The plates were shaken protected from light at 600 – 700 rpm, 4 °C in Titramax shaker (Heidolph) for 1 hour. Plates were washed twice with 200 μL FACS buffer and centrifuged at 300 x g, 4 °C for 5 min after each wash step to remove the supernatant. The cells were subsequently stained with one of two alternative secondary antibodies in FACS buffer: R-Phycoerythrin AffiniPure Goat Anti-Mouse IgG2a Fcg Fragment Specific (Jackson ImmunoResearch, #115-117-186) or R-Phycoerythrin AffiniPure Goat Anti-Human IgG (Jackson ImmunoResearch, #109-115-098). The mixtures were shaken at 600 – 700 rpm, 4 °C for 1 hour. Plates were washed twice with 200 μL FACS buffer and centrifuged at 300 x g, 4 °C for 5 min after each wash step to remove the supernatant. The cells were resuspended in 120 μL FACS buffer containing 1:1,000 SYTOX Blue Dead Cell Stain (Molecular Probes, #S34857) and analyzed using a BD FACSCanto™ flow cytometry system (BD Biosciences).

The *in vitro* binding of the anti-human CCR8 antibodies (BAY 3375968, BAY 3353497, or fully Fc-silenced anti-human CCR8 LALA-aglycosylated variant) and the respective non-binding hIgG1 isotype control antibodies were studied using human CCR8-transfected CHO cells, and CHO cells transfected with empty vector as well as human CCR8-transfected HEK293 cells and HEK293 cells transfected with empty vector.

Adherent cells were harvested non-enzymatically (Cell Dissociation Buffer, enzyme-free, PBS; Gibco, #13151-014) and the cells (100,000 cells/well) were suspended in 100 μL FACS Buffer (auto MACS Rinsing Solution, Miltenyi #130-091-222) + 1:20 MACS BSA Stock Solution (Miltenyi #130-091-376), and stained with the primary antibodies (0.0256, 0.128, 0.64, 3.2, 16, 80, 400, 2000, 10000, 50000, 250000 ng/ml) in 96-well V-Bottom microtiter plates (Falcon, #353263). The plates were shaken protected from light at 600 – 700 rpm, 4 °C in Titramax shaker (Heidolph) for 1 hour. Plates were washed twice with 200 μL FACS buffer and centrifuged at 300 x g, 4 °C for 5 min after each wash step to remove the supernatant. The cells were subsequently stained with secondary antibody in FACS buffer: R-Phycoerythrin AffiniPure Goat Anti-Human IgG (Jackson ImmunoResearch, #109-115-098). The mixtures were shaken at 600 – 700 rpm, 4 °C for 1 hour. Plates were washed twice with 200 μL FACS buffer and centrifuged at 300 x g, 4 °C for 5 min after each wash step to remove the supernatant. The cells were resuspended in 120 μL FACS buffer containing 1:1,000 SYTOX Blue Dead Cell Stain (Molecular Probes, #S34857) and analyzed using a BD FACSCanto™ flow cytometry system (BD Biosciences).

**ADCC and ADCP activities of anti-mouse CCR8 antibodies *in vitro***

The antibody-dependent cellular cytotoxicity (ADCC) and antibody-dependent cellular phagocytosis (ADCP) activities of the anti-mouse CCR8 antibodies (hIgG1, N297A-aglycosylated hIgG1, mIgG2a) and the respective non-binding isotype control antibodies were determined using mouse CCR8-transfected HEK293 cells as target cells. The effector cells were primary mouse NK cells or primary mouse bone marrow-derived M2 macrophages in newly established ADCC and ADCP experiments, respectively.

Mouse CCR8 expressing HEK293 cells used as target cells were generated at InSCREENeX. The cells were cultured in RPMI 1640 Medium, GlutaMAX™ Supplement (Gibco, #61870-010) supplemented with 10 % fetal calf serum (FCS; Sigma, #F2442) and 1 mg/mL G418 (Corning, #30-234-CR), and maintained at 37 °C in a humidified 5 % CO_2_ incubator.

*Mouse ADCC assessment using mouse CCR8 expressing HEK293 cells as target cells and primary mouse NK cells as effector cells*

For the ADCC assessment, mouse CCR8 expressing HEK293 target cells were stained with 0.5 µM IncuCyte® Cytolight Rapid Red Dye (Sartorius, #4706) at 37 ºC for 2 x 10 min. Cytolight Red is a cytoplasmic label of living target cells that freely passes membranes, and is transferred to daughter cells, but not to adjacent cells. The target cells were washed once with an excess of the ADCC assay medium [RPMI 1640 supplemented with 1% fetal bovine serum (FBS, ultra-low IgG One Shot™; Gibco, #A388 19-01)], and re-suspended at the appropriate cell density in ADCC assay buffer. Viability of the cells was higher than 90 %. A total of 50 μL of the Cytolight Red stained target cells (10,000 cells per well) were transferred to 96-well plates and allowed to settle at room temperature for 30 min. The plates were kept at 37 °C in a humidified 5 % CO_2_ incubator overnight until used in the ADCC assay (approximately 20 hours).

Primary mouse NK cells isolated from 10–12 weeks old female BALB/c mouse spleens were used as effector cells for the ADCC assessments. Mouse spleens were transferred into gentleMACS™ C tubes (Miltenyi Biotec, #130-093-237) and dissociated using Spleen Dissociation Kit (Miltenyi Biotec, #130-095-926 and gentleMACS™ Octo Dissociator including Heaters (Miltenyi Biotec). The cell suspension was applied to a Pre-Separation Filter, (30 μm; Miltenyi Biotec, #130-041-407) to obtain a single-cell suspension. The cells were spun down (300 × *g* for 10 min) and resuspended at a cell density of 10^7^ cells per 40 µl cold isolation buffer [MACS® BSA Stock Solution (Miltenyi Biotec, #130-091-376) diluted 1:20 with autoMACS® Rinsing Solution (Miltenyi Biotec, #130-091-222)]. Subsequently, NK Cell Biotin-Antibody Cocktail (NK Cell Isolation Kit, mouse; Miltenyi Biotec, #130-115-818) was added (10 μL/10^7^ cells), the mixture was incubated at 2 – 8 ºC for 5 min, and the reaction was stopped by adding cold isolation buffer (80 μL/10^7^ cells). After additional adding and incubation with anti-biotin MicroBeads (20 μL/10^7^ cells; NK Cell Isolation Kit, mouse; Miltenyi Biotec, #130-115-818) at 2 – 8 ºC for 10 min, the cell suspension was loaded onto MACS LS column (Miltenyi Biotec, #130-042-401, prepared by rinsing with 3ml of cold isolation buffer) and the flow-through containing the unlabeled enriched NK cells were collected, spun down, and resuspended in ADCC assay medium. For the ADCC assessment, freshly isolated mouse NK cells were pre-stimulated with a recombinant mouse IL-15 protein (rmIL-15; R&D System, #447-ML-010/CF) at 1 ng/mL in the ADCC assay medium at 37 °C in a humidified 5 % CO_2_ incubator for 24 hours. After stimulation, NK cells were washed once with ADCC assay medium, and re-suspended at the appropriate cell density in ADCC assay medium. Viability of the cells used in the ADCC assay was higher than 90 %. Both target and effector cells were suspended at the appropriate cell densities in ADCC assay medium. The 96-well plate containing 50 μL of the Cytolight Red stained target cells (10,000 cells per well) were taken out of the incubator and 50 μL of Incucyte® Caspase-3/7 Green Dye for Apoptosis (diluted 1:250 in ADCC medium; Sartorius, #4440) was added to the wells. Caspase-3/7 Green is an inert, non-fluorescent substrate that freely crosses the cell membrane where it can be cleaved by activated caspase-3/7, and subsequently labels fluorescently the DNA of apoptotic cells. Cytolight Red stained target cells with caspase 3/7 dye were pre-incubated together with 50 μL of various concentrations of the antibodies diluted in ADCC medium (in dose-response study, 10 to 0.0001 μg/mL) at 37 °C for 15 min. Subsequently, 50 μL of effector NK cells were added at the effector-to-target (E:T) ratio 10:1 and real-time cell imaging was performed for 72 hours using the Incucyte® S3 Live-Cell Analysis System (Sartorius). Digitonin (0.15 %; Promega, #G9441) was used as a positive control to determine maximum lysis of target cells. Percentage of cytotoxicity was calculated in relation to no-antibody control using the formula where ADCC % = [(experimental value – no-antibody control) / (maximal lysis of target cells – spontaneous lysis of target cells)] x 100 %.

*Mouse ADCP assessment using mouse CCR8 expressing HEK293 cells as target cells and mouse M2 macrophages as effector cells*

Mouse M2 bone marrow-derived macrophages (BMDM) were used as effector cells for the mouse ADCP assessments. Mouse (C57BL/6N; Charles River) bone marrow cells were isolated from murine long bones (femurs and tibiae), spun down (1,200 rpm for 7 min), resuspended in 1 mL of 1 x lysis buffer (Red Blood Cell Lysing Buffer Hybri-Max™; Sigma, # R7767), and incubated at room temperature for 7 min. Subsequently, the cells were washed twice with medium [DMEM/F‑12, GlutaMAX™ Supplement (Gibco, #31331-028) containing 10 % heat-inactivated fetal calf serum (FCS; Sigma, #S0615) and 1% Penicillin-Streptomycin (Gibco, #15140-122) and resuspended at the appropriate cell density (viability of the cells was 93 %) in differentiation medium containing 20 ng/mL macrophage colony-stimulating factor (M‑CSF; BioLegend, #576406). The primary bone marrow cells were seeded onto 96-well plates (40,000 cells/well) and incubated at 37 °C in a humidified 5 % CO_2_ incubator for 6 days (after 3 days, half of the medium was exchanged with fresh medium). On day 6, the cells were polarized into M2 macrophage subset with 20 ng/mL M‑CSF, 50 ng/mL recombinant mouse interleukin-4 (rmIL-4; BioLegend, #574306) and 50 ng/ml rmIL-13 (BioLegend, #575906) stimulation for 1 day. For the ADCP assessment, mouse CCR8 expressing HEK293 target cells were stained with 333 nM IncuCyte® Cytolight Rapid Green Dye (Sartorius, #4705) labeling all live cells and 0.5 μg/mL IncuCyte® pHrodo™ Red Cell Labeling Dye from the IncuCyte® pHrodo™ Red Cell Labeling Kit for Phagocytosis (Sartorius, #4649) for labeling cells with a pH-sensitive fluorophore according to manufacturer’s instructions to label phagocytosed target cells.

Briefly, the target cells were washed once with 1 mL Dulbecco’s phosphate-buffered saline (DPBS) per 1 x 10^6^ cells, resuspended at 1 x 10^6^ cells/ mL concentration in DPBS and incubated with 333 nM of IncuCyte® Cytolight Rapid Green Dye at 37 °C for 20 min. Excess dye was bonded by adding a 6-fold volume of serum-containing cell culture medium and waiting for 5 min. Cells were spun down (500 x g for 5 min) washed once with 1 mL of IncuCyte® pHrodo™ Wash Buffer (component C) per 1 x 10^6^ cells, resuspended at 1 x 10^6^ cells/ mL concentration in IncuCyte® pHrodo™ Red Labeling Buffer (component D), and incubated with 0.5 μg/mL of IncuCyte® pHrodo™ Red Cell Labeling Dye [(component A diluted in DMSO (component B)] at 37 °C for 60 min. The cells were spun down (1,000 rpm for 7 min), washed once with the ADCP assay medium without cytokines, and resuspended at the appropriate cell density in ADCP assay medium [DMEM/F-12, GlutaMAX™ Supplement (Gibco, #31331-028) supplemented with 10% fetal bovine serum (FBS; Sigma, #S0615), 20 ng/mL M-CSF, 50 ng/mL rmIL-4, 50 ng/mL rmIL‑13, and 1 % Penicillin-Streptomycin]. Viability of the cells used in the ADCP assay was 97 %. The studied antibodies at 0.00064, 0.0032, 0.016, 0.08, 0.4, 2, and 10 μg/mL, diluted in ADCP assay medium, and the IncuCyte® pHrodo™ Red-labeled target cells were added at effector-to-target (E:T) ratio of 4:1 on the 96-well plates containing the effector cells (mouse M2 macrophages; 40,000 cells per well) and the plates were incubated at 37 °C in a humidified 5 % CO_2_ incubator. After 30 min of pre-incubation, the plates were scanned in phase, green and red fluorescence channel using the Incucyte® S3 Live-Cell Analysis System (Sartorius). Thereafter, the scanning was performed every 60 min for 12 hours long co-culturing time period.

Cumulative phagocytosis measures the percentage of target cells removed until the given timepoint, and it was calculated using the formula: ADCP % = 100 % – [Experimental value (green signal of antibody + target cells + effector cells) / background value (green signal of no antibody control target cells + effector cells) x 100 %]. Real-time phagocytosis measures the percentage of target cells being phagocytosed at a given timepoint, and it was calculated using the formula: ADCP % = Experimental value [(green and red signal of antibody + target cells + effector cells) / green signal] – background value [(green and red signal of no antibody control target cells + effector cells) / green signal] x 100 %. Red indicates phagocytosed target cells and green all target cells.

**ADCC and ADCP activities of anti-human CCR8 antibodies *in vitro***

The human ADCC and ADCP activities of afucosylated anti-human CCR8 antibody BAY 3375968, its conventionally glycosylated variant BAY 3353497, its fully Fc-silenced LALA-aglycosylated variant, and the respective isotype controls were determined using different newly established human ADCC and ADCP settings.

In some human ADCC and ADCP experiments, human primary Tregs were used as target cells, while in others human CCR8 expressing HEK293 cells were used as target cells. The human Tregs were obtained from peripheral blood mononuclear cells (PBMCs) from two donors, stimulated and their CCR8 expression levels were confirmed by flow cytometry. Human CCR8 expressing HEK293 cells were generated by InSCREENex GmbH) and cultured in DMEM, high glucose, GlutaMAX™ Supplement, pyruvate (Gibco, #31966-021) supplemented with 10 % fetal bovine serum (FBS) and 1 mg/mL G418 (Alfa Aesar, #J63871). For the ADCC and ADCP assessment, human CCR8 expressing HEK293 target cells were sorted by flow cytometry to get the 10 % of cells with the highest CCR8 expression level.

As effector cells either human NK92v cells or primary human NK cells were used in the ADCC experiments whereas human M2c macrophages were used in the ADCP experiments.

*Human ADCC assessment using primary human Tregs as target cells and NK92v cells as effector cells*

Human Tregs used as target cells were isolated from PBMCs of healthy donors. Expression levels of CCR8 in the *in vitro*-activated Tregs were confirmed by flow cytometry. The human Tregs were cultured in 1640 RPMI (Gibco, #11875-093) supplemented with 10 % fetal bovine serum (FBS), 1x Basal Medium Eagle (BME), 1 % Na-pyruvate, 1 % non-essential amino acids (NEAA), 1 % L-glutamine (GlutaMAX™), 1 % HEPES, and 500 IU/mL recombinant interleukin-2 (rIL-2; R&D Systems). The cells were maintained at 37 °C in a humidified 5 % CO_2_ incubator. Viability of the target cells was approximately 90 %. For analysis of the expression levels of CCR8 on the activated human Tregs, the cells were washed two times with FACS buffer [1x PBS (Thermo Fisher), 2 mM EDTA, 0.1 % bovine serum albumin (BSA)] before seeded onto 96-well round-bottom plates (1 x 10^5^ cells/well). Subsequently, the cells were incubated with 0.5 mg/mL APC-labelled anti-CCR8 antibody (clone L263G8; BioLegend) or isotype control (mIgG2a; BioLegend) on ice for 30 min, washed two times with FACS buffer, and resuspended in 200 µL of FACS buffer. Dead cells were excluded by staining with 7-amino-actinomycin D (7-AAD; Miltenyi Biotec) prior to the analysis. The flow cytometric analysis was performed using MACSQuant® Analyzer (Miltenyi Biotec).

NK92v (158V) cells (NantKwest) were used as effector cells for these ADCC assessments. NK92v is a NK-like cell line, engineered to express the high affinity hFcγRIIIA activating receptor [1]. The cells were cultured in MyeloCult™ H5100 (StemCell Technologies) supplemented with 100 IU/mL recombinant human interleukin-2 (rhIL-2; Miltenyi Biotec, #130-097-745), and 1 µM hydrocortisone (StemCell Technologies, #07904). The cells were maintained at 37 °C in a humidified 5 % CO_2_ incubator. Viability of the cells ranged from 77 % to 88 %.

Target and effector cells were washed once with warm ADCC assay medium (RPMI 1640 supplemented with 1 % FBS, 100 IU/mL penicillin/ streptomycin, 1 mM Na-pyruvate, and 1 x NEAA) and resuspended at the appropriate cell densities. Target cells and effector cells were seeded at effector-to-target (E:T) ratio 4:1 onto 96-well plates (Corning® 96-well Flat Clear Bottom White Polystyrene TC-treated Microplates, #3903) containing various concentration of the antibodies (0.0001–1 μg/mL). The plates were incubated at 37 °C for 2 hours. Subsequently, AAF-Glo™ Substrate (Promega CytoTox-Glo™ Cytotoxicity Assay kit; Promega Corporation, #G9292) was added, the plates were covered in foil and shaken at room temperature for 15 min, and the luminescence was measured using the CytoTox-Glo program on GloMax®-Multi+ Microplate Reader. Digitonin (Promega Corporation, #G9448) was used as a positive control (maximal lysis). Percentage cytotoxicity was calculated using the formula = [(experimental value – no antibody control) / (maximal lysis of target cells – spontaneous lysis of target cells)] x 100 %.

*Human ADCC assessment using human CCR8 expressing HEK293 cells as target cells and NK92v cells or primary human NK cell as effector cells*

In some experiments NK92v (158V) cells (NantKwest) were used as effector cells for ADCC assessments. NK92v is a NK-like cell line, engineered to express the high affinity hFcγRIIIA activating receptor [1]. The cells were cultured in MyeloCult™ H5100 (StemCell Technologies) supplemented with 100 IU/mL recombinant human interleukin-2 (rhIL-2; Miltenyi Biotec, #130-097-745), and 1 µM hydrocortisone (StemCell Technologies, #07904). The cells were maintained at 37 °C in a humidified 5 % CO_2_ incubator. Viability of the cells was above 90 %.

In other experiments primary human NK cells were used as effector cells for ADCC assessments. Primary human NK cells were isolated from PBMCs obtained from citrated whole blood of human healthy donors by gradient centrifugation using Pancoll (PAN Biotech, #P04-66500). Isolated NK cell population was verified by CD3/CD57 and CD16, CD32, CD64 expression levels via flow cytometry. For primary human NK cell isolation, PBMC cells were resuspended at a cell density of 10^7^ cells per 40 µl cold isolation buffer [MACS® BSA Stock Solution (Miltenyi Biotec, #130-091-376) diluted 1:20 with autoMACS® Rinsing Solution (Miltenyi Biotec, #130-091-222)] and Biotin-Antibody Cocktail (NK Cell Isolation Kit, human; Miltenyi Biotec, #130-092-657) was added in a volume of 10 μL per 10^7^ total PBMC cells. The cell suspension was incubated at 2 – 8 ºC for 5 min and reaction was stopped by addition of 30 µl cold isolation buffer per initial 10^7^ PBMC cells. Subsequently, NK Cell MicroBead Cocktail (20 μL/10^7^ cells; NK Cell Isolation Kit, human; Miltenyi Biotec, #130-092-657) was added and incubated at 2 – 8 ºC for 10 min, followed by loading suspension onto the MACS LS column (Miltenyi Biotec, #130-042-401, prepared by rinsing with 3 ml of cold isolation buffer). The flow-through, containing unlabelled enriched NK cells, were collected, spun down, resuspended in RPMI 1640 (Gibco, #61870-036) containing 10 % FBS and maintained at 37 °C in a humidified 5 % CO2 incubator for 20 hours.

For ADCC Assay, 30,000 human CCR8 expressing HEK293 cells per well were seeded in 50 µl ADCC assay medium (Gibco # 61870036 RPMI 1640, supplemented with 1 % FBS, 1 mM Na-pyruvate, and 1 x NEAA) by using 96-well U-bottom plates (Falcon, #353077). 50 µl of various antibody dilutions (0.0000001 µg/ml – 10 µg/ml, diluted in ADCC assay medium) was added and pre-incubated at 37 °C for 30 min in a humidified 5 % CO2 incubator. The effector cells NK92v or primary human NK cells were washed once in ADCC assay medium and added in an effector-to-target (E:T) ratio of 4:1 by using a volume of 50 µl/well. For calculation of relative cytotoxicity, target cells only were treated with 1 % Triton™ X‑100 (polyethylene glycol tert-octylphenyl ether, Sigma, #93443-100ml, 10 % solution in H2O) to get maximum target cell lysis. Additionally, no antibody controls (effector and target cells only) and target cell only controls were included. After all components and controls have been pipetted together, 4h incubation was started at 37 °C with 5 % CO2 in a humidified incubator. For lactate dehydrogenase (LDH) release, cells were spun down at 250 x g for 5 min and 90 µl of the supernatants were transferred to 96-V bottom plates (Corning, #3894), followed by a second centrifugation step at 300 x g for 5 min. 50 µl of the supernatant were transferred to 96-well flat-bottom analysis plates (Corning,#3599) and incubated with 50 µl of reconstituted Cytotoxicity Detection reagent for 30 min in dark (without shaking). Cytotoxicity Detection reagent [Cytotoxicity Detection Kit (LDH) Roche, #11644793001] was prepared by reconstitution the lyophilizate by adding of 1ml doubled distilled water for 10 minutes (=Catalyst), followed by 1:46 dilution with containing dye solution. Reaction was stopped by adding 50 µl of 0,6 mol/l Hydrochloric Acid (Merck; #1.09057.1000) and lactate dehydrogenase (LDH) release was measured at 490 nm with reference 630 nm at Tecan SPARK instrument.

Percentage of cytotoxicity was calculated in relation to no-antibody control using the formula where ADCC % = [(experimental value – no-antibody control) / (maximal lysis of target cells – spontaneous lysis of target cells)] x 100 %.

*Human ADCP assessment using primary human Tregs as target cells and human M2 macrophages as effector cells*

Human Tregs used as target cells were isolated from PBMCs of healthy donors. Expression levels of CCR8 in the *in vitro*-activated Tregs were confirmed by flow cytometry. The human Tregs were cultured in 1640 RPMI (Gibco, #11875-093) supplemented with 10% fetal bovine serum (FBS), 1x Basal Medium Eagle (BME), 1 % Na-pyruvate, 1 % non-essential amino acids (NEAA), 1 % L-glutamine (GlutaMAX™), 1 % HEPES, and 500 IU/mL recombinant interleukin-2 (rIL-2; R&D Systems). The cells were maintained at 37 °C in a humidified 5 % CO_2_ incubator. Viability of the target cells was approximately 90 %. For analysis of the expression levels of CCR8 on the activated human Tregs, the cells were washed two times with FACS buffer [1x PBS (Thermo Fisher), 2 mM EDTA, 0.1 % bovine serum albumin (BSA)] before seeded onto 96-well round-bottom plates (1 x 10^5^ cells/well). Subsequently, the cells were incubated with 0.5 mg/mL APC-labelled anti-CCR8 antibody (clone L263G8; BioLegend) or isotype control (mIgG2a; BioLegend) on ice for 30 min, washed two times with FACS buffer, and resuspended in 200 µL of FACS buffer. Dead cells were excluded by staining with 7-amino-actinomycin D (7-AAD; Miltenyi Biotec) prior to the analysis. The flow cytometric analysis was performed using MACSQuant® Analyzer (Miltenyi Biotec).

Human M2c macrophages differentiated from human blood monocytes were used as effector cells for these ADCP assessments. Human blood monocytes were isolated from PBMCs of healthy donors by using the Pan Monocyte Isolation Kit (Miltenyi, #130-096-537) and LS Columns (Miltenyi, #130-042-401) according to the manufacturer’s instructions. The isolated primary monocytes were seeded onto culture dishes (Corning, #430591) and cultured in ImmunoCult™-SF Macrophage Medium [StemCell Technologies, #10961; supplemented with 50 ng/mL recombinant human M-CSF (Stem Cell Technologies; #78057)] for 5 days (after 3 days fresh medium was added). Subsequently, the macrophages were polarized into M2c subset with 10 ng/mL recombinant human interleukin-10 (rhIL-10; Peprotech, #AF‑200-10) stimulation for 2 days.

Media was collected in pre-chilled conical tubes. The IL-10-activated macrophages were rinsed once with Dulbecco’s phosphate-buffered saline (DPBS), 5 mL of 2.5 mM EDTA in DPBS was added to each culture dish, the dishes were incubated at 37 °C for 10 min, and the cell suspensions were transferred with a pipet to pre-chilled conical tubes. Subsequently, 5 mL PBS + bovine serum albumin (BSA) was added to each dish and transferred a few seconds later to the chilled conical tubes with the collected cell suspension. All dishes were quickly rinsed with the same 10 mL of PBS and the rinse was transferred to the pre-chilled tubes with the collected cells. The tubes were centrifuged at 1,500 rpm at 4 °C for 5 min and the M2c macrophages were resuspended in cold X-Vivo™ 15 medium. Viability of the cells prior to the ADCP assay was 96 %.

For the ADCP assay, human primary Treg cells as target cells were incubated with CFSE labeling solution (Thermo Fisher, #C34554) at 1 x 10^6^ cells/mL at 37 °C for 15 min with gentle mixing. Subsequently, the cells were washed twice with X-Vivo™ 15 medium (Lonza, #04-418Q) to quench the reaction and to remove free CFSE label, and the cells were resuspended to 400,000 cells/mL in X-Vivo™ 15 medium. Effector cells and CFSE- labeled target cells were seeded at effector-to-target (E:T) ratio 4:1 onto sterile low-binding round-bottom 96-well assay plates, and the plates were incubated at 37 °C for 4 hours. Subsequently, the cell suspension was transferred to a clean 96-well FACS plate. Some of the cells remained attached to the wells and 150 μL of cold MACS buffer was added to the wells to allow for a later transfer of the remaining cells to the FACS plates.

The cells transferred to the FACS plates in the first transferring step were spun down (2,000 rpm at 4 °C for 2 min) and the media was removed. The MACS buffer with the remaining cells from the original assay plates was used to resuspend the cell pellets. The cells from the combined cell mixture were spun down, FcR block solution (Miltenyi, #130-059-901) was added to prevent non-specific binding, and the plates were incubated on ice for 10 min. APC-conjugated anti-CD206 or iso‑APC control antibody was added to the wells and the plates were incubated on ice for 30 min. Subsequently, the cells were pelleted and washed twice with 150 μL cold MACS buffer and resuspended in 75 μL cold MACS buffer containing 7-amino-actinomycin D (7-AAD; Miltenyi Biotec) to exclude dead cells. The flow cytometric analysis of phagocytosis of CFSE-labeled target cells by APC-positive M2c macrophages was performed using MACSQuant® Analyzer (Miltenyi Biotec). Phagocytosis was determined as % of CFSE+ CD206+ double-positive macrophages, i.e. providing the percentage of macrophages that are phagocyting.

*Human ADCP assessment using human CCR8 expressing HEK293 cells as target cells and human M2 macrophages as effector cells*

Human M2c macrophages differentiated from human blood monocytes were used as effector cells for these ADCP assessments. Human blood monocytes were isolated from PBMCs of healthy donors by using the Pan Monocyte Isolation Kit (Miltenyi, #130-096-537) and LS Columns (Miltenyi, #130-042-401) according to the manufacturer’s instructions. The isolated primary monocytes were cultivated in differentiation medium Cell-X-Vivo 15 [Lonza, #BE02-060F; supplemented with 50 ng/mL M-CSF (BioLegend, #574806), seeded onto ultra-low attachment 6-well plates (Corning, #3471) (1E06 cells/mL) and incubated at 37 °C in a humidified 5 % CO_2_ incubator for 5 days (after 2 days, same volume of fresh medium was added). Subsequently, the macrophages were polarized into M2c subset with 10 ng/mL recombinant human interleukin-10 (BioLegend, #571004) stimulation for 2 days.

Media from all wells with the IL-10-activated macrophages were collected in 50 ml tubes. Immediately ice-cold Dulbecco’s phosphate-buffered saline (DPBS) was added to the cells and then incubated for 15-20 min at 4 °C. Cells were resuspended and by thus detached from the bottom of the 6-well plate and collected in 50 ml tubes. The tubes were centrifuged at 300 x g at 4 °C for 10 min and the M2c macrophages were resuspended in Cell-X-Vivo 15 [Lonza, #BE02-060F; supplemented with 50 ng/mL M-CSF (BioLegend, #574806) and 10 ng/ml IL-10 (BioLegend, #571004)]. After counting with Countess II M2c macrophages were seeded onto 96-well plates (40,000 cells/well; Corning, #3599) and incubated at 37 °C in a humidified 5 % CO_2_ incubator.

For the ADCP assessment, human CCR8 expressing HEK293 target cells (high CCR8 expression) were stained with 333 nM IncuCyte® Cytolight Rapid Green Dye (Sartorius, #4705) labeling all live cells and 0.5 μg/mL IncuCyte® pHrodo™ Red Cell Labeling Dye from the IncuCyte® pHrodo™ Red Cell Labeling Kit for Phagocytosis (Sartorius, #4649) for visualizing phagocytosed cells with a pH-sensitive fluorophore according to manufacturer’s instructions.

Briefly, the human CCR8 expressing HEK293 target cells were washed once with 1 mL DPBS per 1 x 10^6^ cells, resuspended at 1 x 10^6^ cells/ mL concentration in DPBS and incubated with 333 nM of IncuCyte® Cytolight Rapid Green Dye at 37 °C for 20 min. Excess dye was bonded by adding a 6-fold volume of serum-containing cell culture medium and waiting for 5 min. Cells were spun down (500 x g for 5 min), washed once with 1 mL of IncuCyte® pHrodo™ Wash Buffer (component C) per 1 x 10^6^ cells, resuspended at 1 x 10^6^ cells/ mL concentration in IncuCyte® pHrodo™ Red Labeling Buffer (component D), and incubated with 0.5 μg/mL of IncuCyte® pHrodo™ Red Cell Labeling Dye [(component A diluted in DMSO (component B)] at 37 °C for 60 min. The cells were spun down (1,000 rpm for 7 min), washed once with serum-containing cell culture medium, and resuspended at the appropriate cell density in ADCP assay medium [Lonza, #BE02-060F; supplemented with 50 ng/mL M-CSF (BioLegend, #574806) and 10 ng/ml IL-10 (BioLegend, #571004)]. Viability of the cells used in the ADCP assay was 96 %. The studied antibodies at 0.00032, 0.0016, 0.008, 0.04, 0.2 and 1 μg/mL, diluted in ADCP assay medium, and the green- and red-labeled target cells were added at effector-to-target (E:T) ratio of 10:1 on the 96-well plates containing the effector cells (human M2 macrophages; 40,000 cells per well) and the plates were incubated at 37 °C in a humidified 5 % CO_2_ incubator. After 30 min of pre-incubation, the plates were scanned in phase, green and red fluorescence channel using the Incucyte® S3 Live-Cell Analysis System (Sartorius). Thereafter, the scanning was performed every 2 hours until the last scanning time at 48 hours.

Cumulative phagocytosis measures the percentage of target cells removed until the given timepoint, and it was calculated using the formula: ADCP % = 100 % – [Experimental value (green signal of antibody + target cells + effector cells) / background value (green signal of no antibody control target cells + effector cells) x 100 %]. Real-time phagocytosis measures the percentage of target cells being phagocytosed at a given timepoint, and it was calculated using the formula: ADCP % = Experimental value [(green and red signal of antibody + target cells + effector cells) / green signal] – background value [(green and red signal of no antibody control target cells + effector cells) / green signal] x 100 %. Red indicates phagocytosed target cells and green all target cells.

**Induction of CDC anti-human CCR8 antibodies *in vitro***

Treg cells were isolated from human PBMCs of healthy donors (n=2), and subsequently expanded and activated *in vitro* to induce CCR8 expression. Therefore, T cells were isolated from frozen PBMC using magnetic-based EasySep™ Human CD4+ CD127^low^ CD25+ Regulatory T Cell Isolation Kit, following the recommended protocol from manufacturer (Stem Cell Technologies # 18063). Treg are defined as CD4+ CD25+ FoxP3+ population in PBMC. Since FoxP3 is an intracellular target that involves fixation and permeabilization in handling, it is not feasible to use FoxP3 as a marker during Treg cell isolation process itself and therefore the isolation kit relies on CD127 due to correlation of CD127 and FoxP3 expression.

Isolated CD4+ CD25+ CD127low Treg cells were expanded for 7 days in TexMACS medium (Miltenyi Biotec #130-097-196) supplemented with 500IU/mL of IL-2 (Miltenyi Biotec # 130-097-745) and TransACT (contains CD3/CD28 antibodies, Miltenyi Biotec # 130-128-758) for activation. Medium was changed once on Day 3 and the cells were harvested on Day 7 for flow cytometer characterization and CDC assay.

Flow cytometry analysis verified that more than 80 % of CD4+ CD25+ FoxP3+ Treg cells expressed CCR8. These cells were then exposed to CDC-qualified human complement serum (Quidel) and various antibodies in a dose-response manner, including afucosylated hIgG1 anti-CCR8 antibody BAY 3375968, a positive control anti-HLA I antibody (W6/32 clone chimerized to human IgG1), and a negative non-binding hIgG1 isotype control antibody.

Antibody dilutions were prepared using CDC medium (TexMACS supplemented with 500 IU/mL IL-2). Treg cells were seeded in 96-well plate at 100,000 cells per well; antibodies were added to Treg at 5 different concentration dilutions (10, 1, 0.1, 0.01 and 0.001 µg/mL). Lastly, serum was added (10 %) and the plate was incubated at 37 °C for 3 hours. 5 µL per well of 7-AAD was added and after further incubation for 5 min, cells were harvested for flow cytometer analysis.

Cell death induced by CDC is indicated as percentage of 7-AAD-positive events. Negative controls were wells contain Treg cell only (no serum, no antibody) and wells with Treg cells with 10 % serum (no antibody).

**ELISA analysis of IFNγ protein in mouse blood and tumors**

The snap-frozen tumor or blood samples were lysed with MSD Tris Lysis Buffer (MSD, Rockville, MD, USA) and stainless-steel beads (5 mm in diameter) using TissueLyser II (Qiagen, Hilden, Germany). The lysates were centrifuged at 25,000 x g at 4 °C for 20 min, and the IFNγ concentration was determined by ELISA using V PLEX Proinflammatory Panel 1 Mouse Kit (MSD, Rockville, MD, USA) in the supernatants.

**Exposure analyses of anti-mouse CCR8 antibodies in plasma**

Quantitative measurements to determine the antibody concentration in mouse plasma were established at the Gyrolab workstation (Gyrolab). For the anti-mouse CCR8 mIgG2a antibody a biotinylated CCR8 murine peptide was used as capture reagent and an Alexa Fluor 647-lableld anti-mouse IgG was used for detection. For the anti-mouse CCR8 hIgG1 and hIgG1-aglycosylated antibodies the generic PK kit (#P0020499, Gyrolab) was used for quantification.

***In vivo* CD8+ T cell ablation in mice treated with anti-mouse CCR8 antibody**

Female CD8-IRES-DTR-EGFP mice (9–10-week-old, Shanghai Model Organisms Center) were inoculated s.c. with 1 x 10^6^ MC38 murine colon cancer cells on day 0. The transgenic mice express the diphtheria toxin receptor under the control of the CD8A promoter. On day 8 at an average tumor size of 96 mm^3^, treatments with the non-binding isotype control antibody or the anti-mouse CCR8 mIgG2a antibody as monotherapy (both at 10 mg/kg, Q3/4Dx4, i.p.) or in combination with diphtheria toxin (0.015 mg/kg, Q3/4Dx4, i.p.) were started (n= 10 mice/group). Tumor growth was observed, and the study was terminated on day 21.

**Statistical analysis**

Statistical analyses were performed with SAS Enterprise Guide (Version 7.15 HF2). The difference between the groups was evaluated using an Analysis of Variance (ANOVA) model with contrasts and the pairwise differences were estimated from this model. Group was introduced in the statistical model as a categorical factor. P values < 0.05 were considered significant. Where the experiment had repeated measures, Time point and Group*Time point interaction was introduced in the model. Association between parameters was evaluated from a regression model with R-square and estimated parameter effect. The survival time was analyzed using the Kaplan-Meier based log-rank test. Additivity of the combination effects was estimated based on comparing the confidence intervals of the combination group with the expected effect of the mono-therapy treatments.

**REFERENCES IN SUPPLEMENTARY INFORMATION**

1. Whiteside SK, Grant FM, Gyori DS, Conti AG, Imianowski CJ, Kuo P, et al. CCR8 marks highly suppressive Treg cells within tumours but is dispensable for their accumulation and suppressive function. Immunology. 2021;163(4):512-20. doi: 10.1111/imm.13337.
