# Supplementary figures and images for: Selective depletion of tumor-infiltrating regulatory T cells with BAY 3375968, a novel Fc-optimized anti-CCR8 antibody
Source: Clin Exp Med. 2024 Jun 10;24(1):122. doi: 10.1007/s10238-024-01362-8 (PMC11164760; doi:10.1007/s10238-024-01362-8)

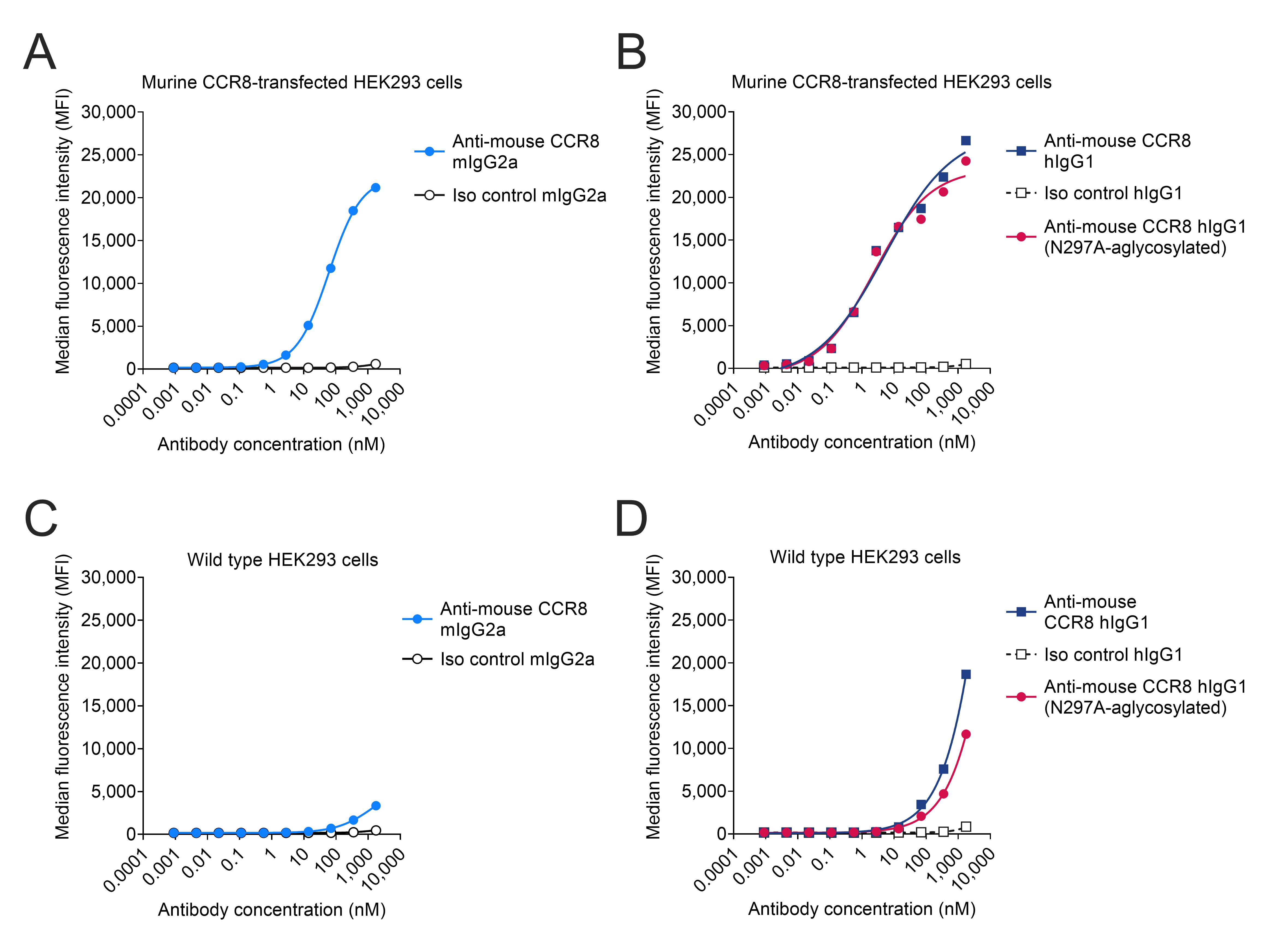

Supplement: Supplementary file 2 — Supplementary file2 (JPG 775 KB) [file 10238_2024_1362_MOESM2_ESM.jpg]

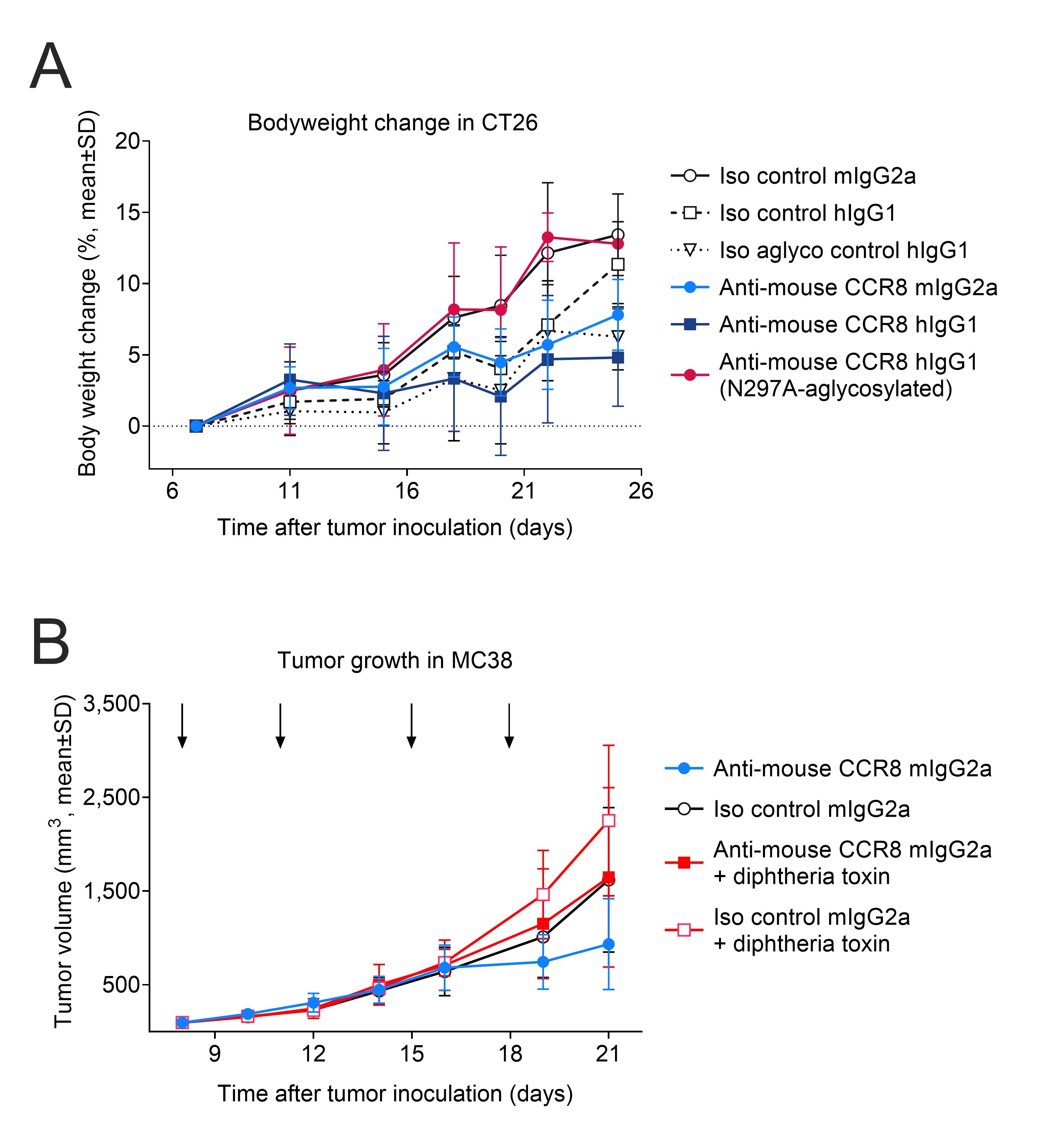

Supplement: Supplementary file 3 — Supplementary file3 (JPG 496 KB) [file 10238_2024_1362_MOESM3_ESM.jpg]

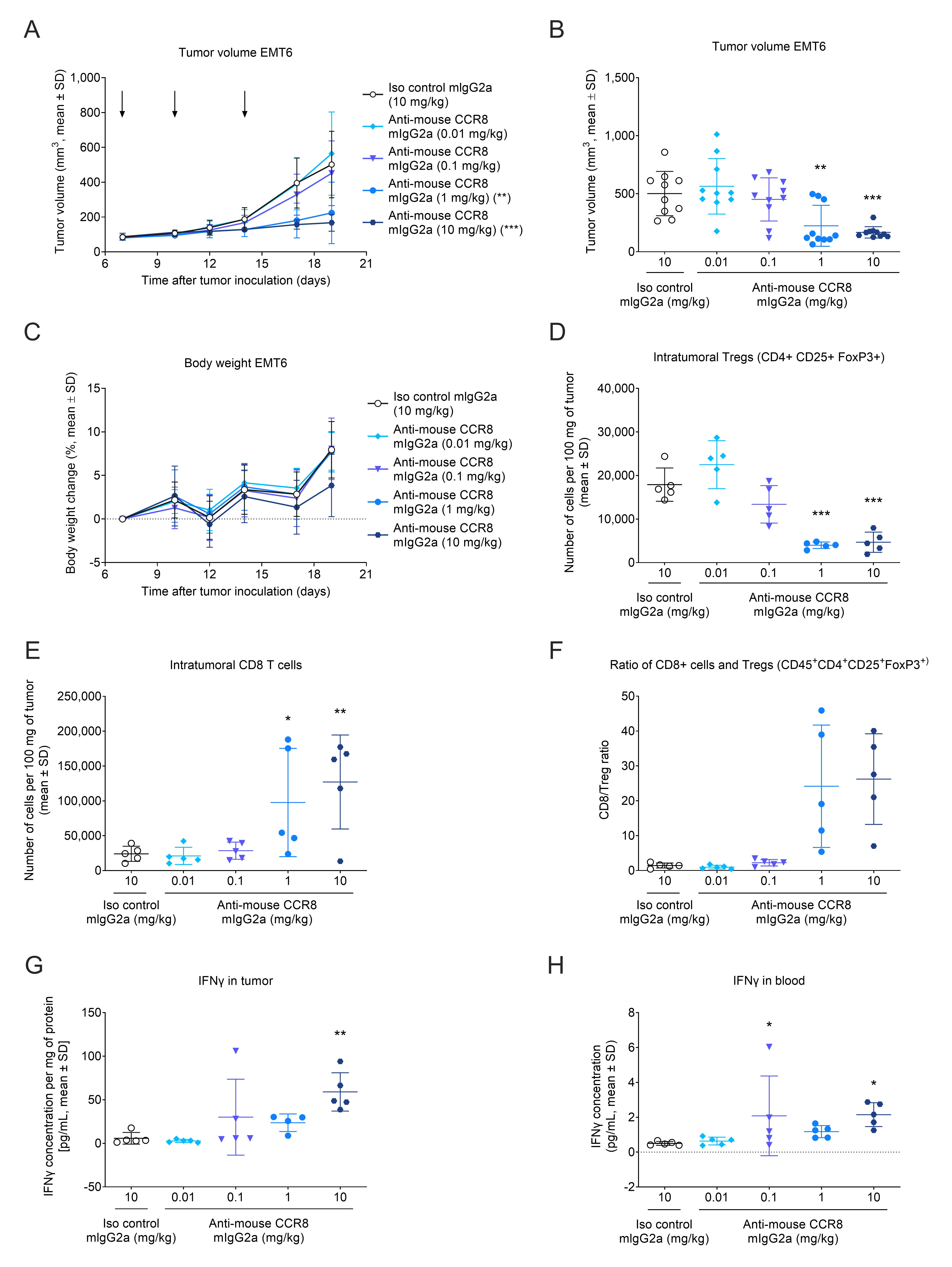

Supplement: Supplementary file 4 — Supplementary file4 (JPG 1518 KB) [file 10238_2024_1362_MOESM4_ESM.jpg]

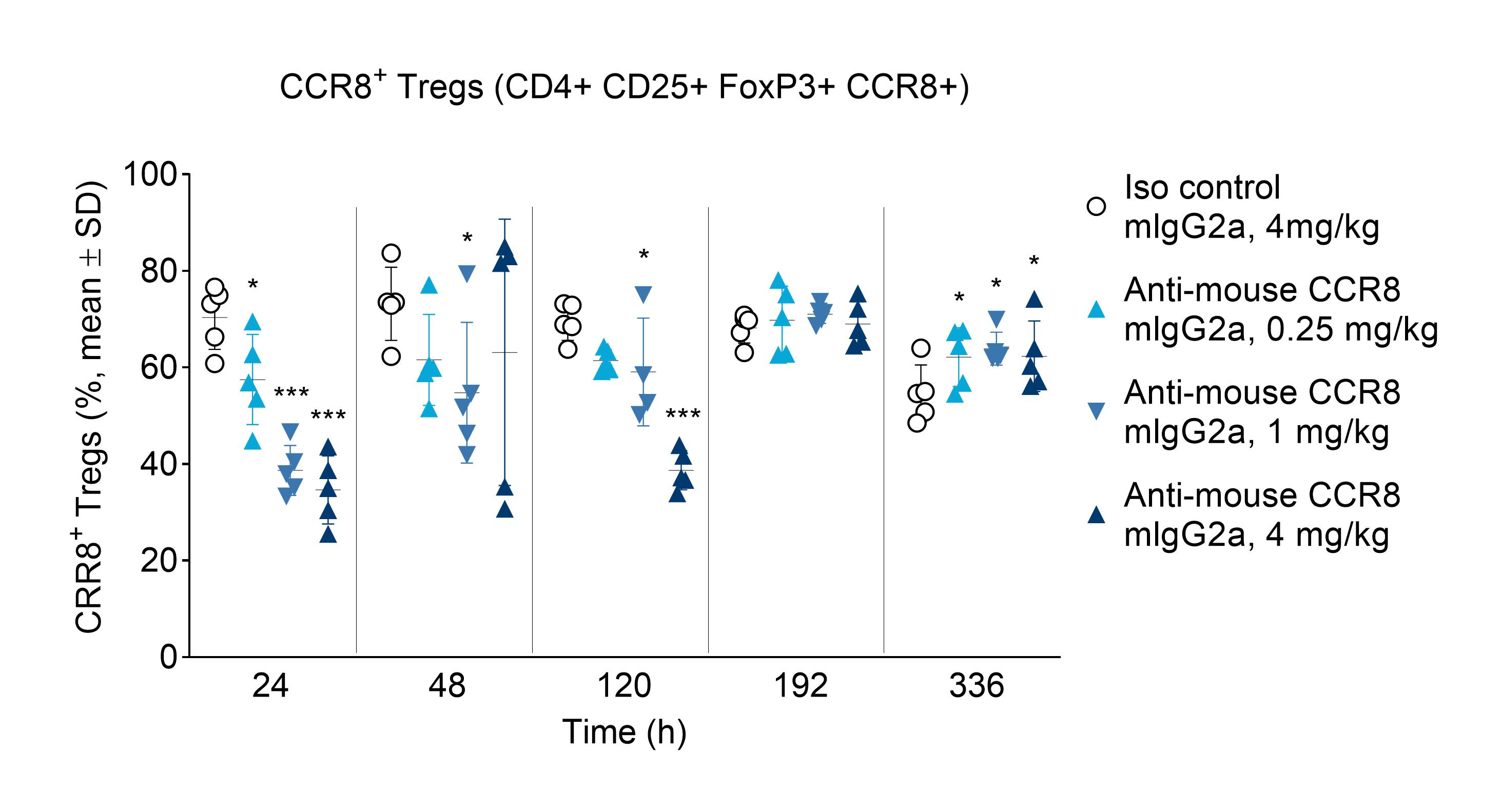

Supplement: Supplementary file 5 — Supplementary file5 (JPG 240 KB) [file 10238_2024_1362_MOESM5_ESM.jpg]

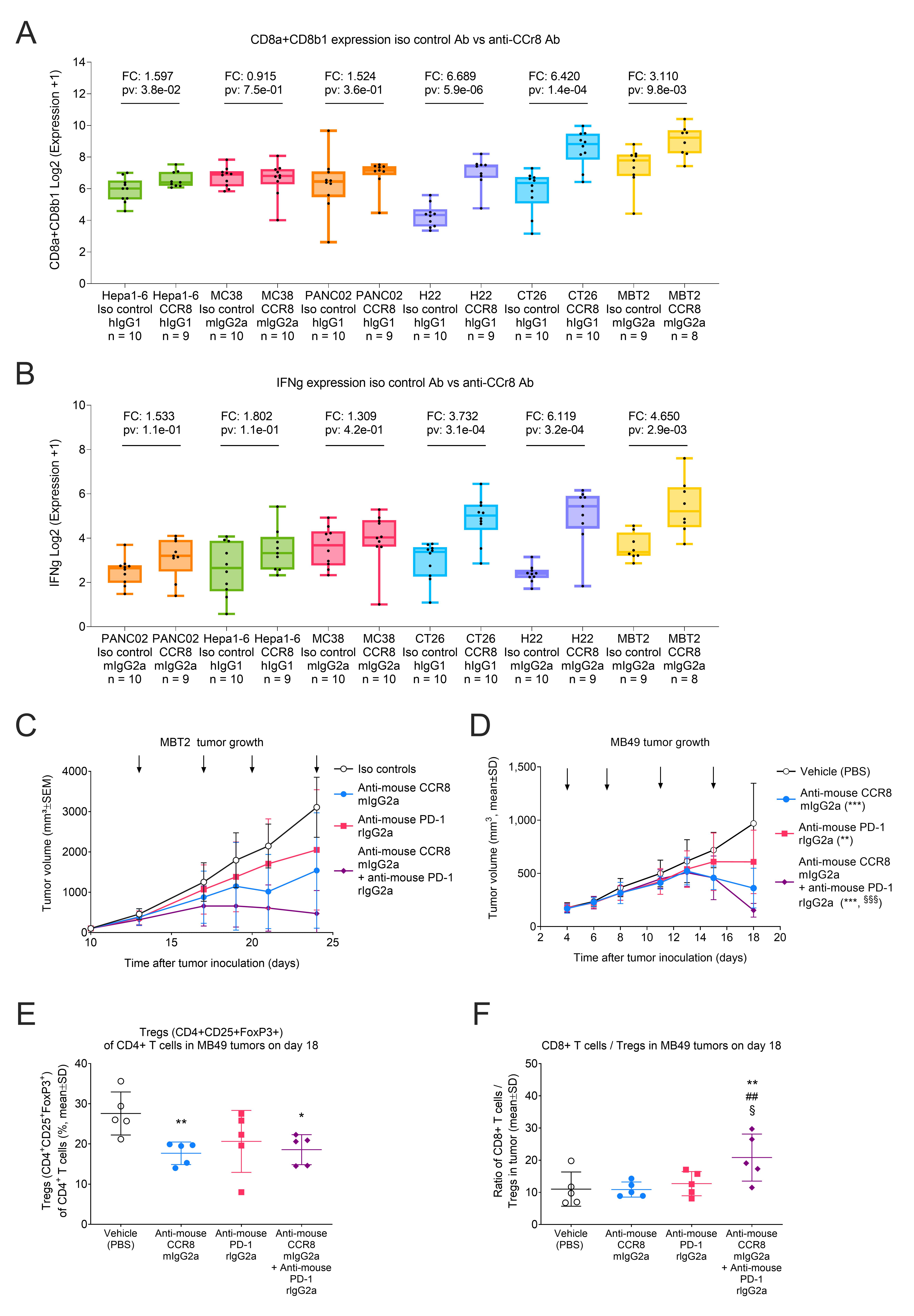

Supplement: Supplementary file 6 — Supplementary file6 (JPG 1824 KB) [file 10238_2024_1362_MOESM6_ESM.jpg]

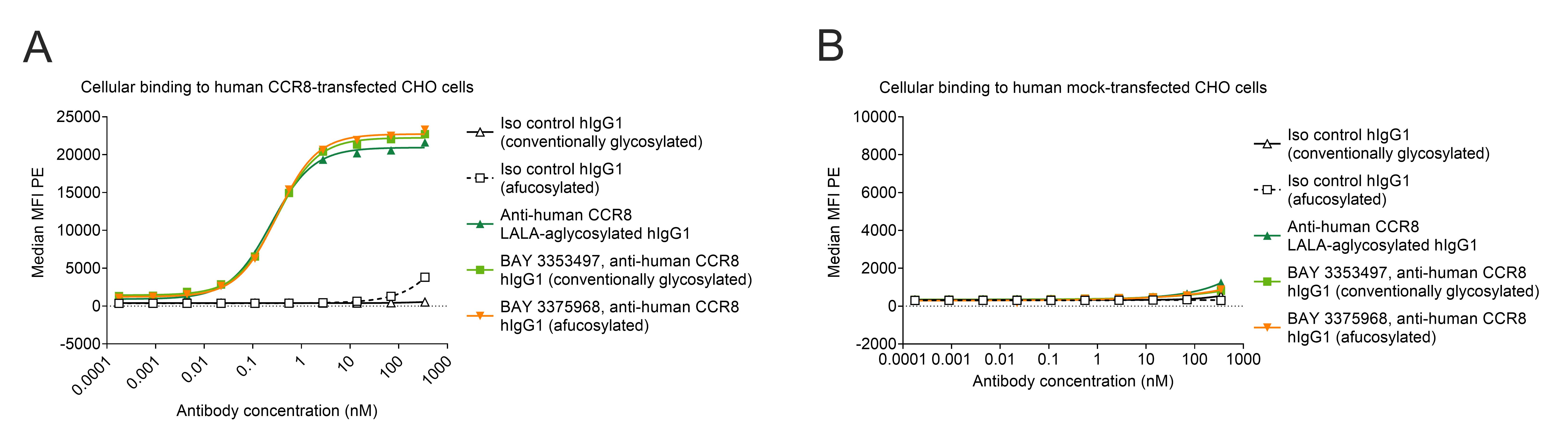

Supplement: Supplementary file 7 — Supplementary file7 (JPG 621 KB) [file 10238_2024_1362_MOESM7_ESM.jpg]

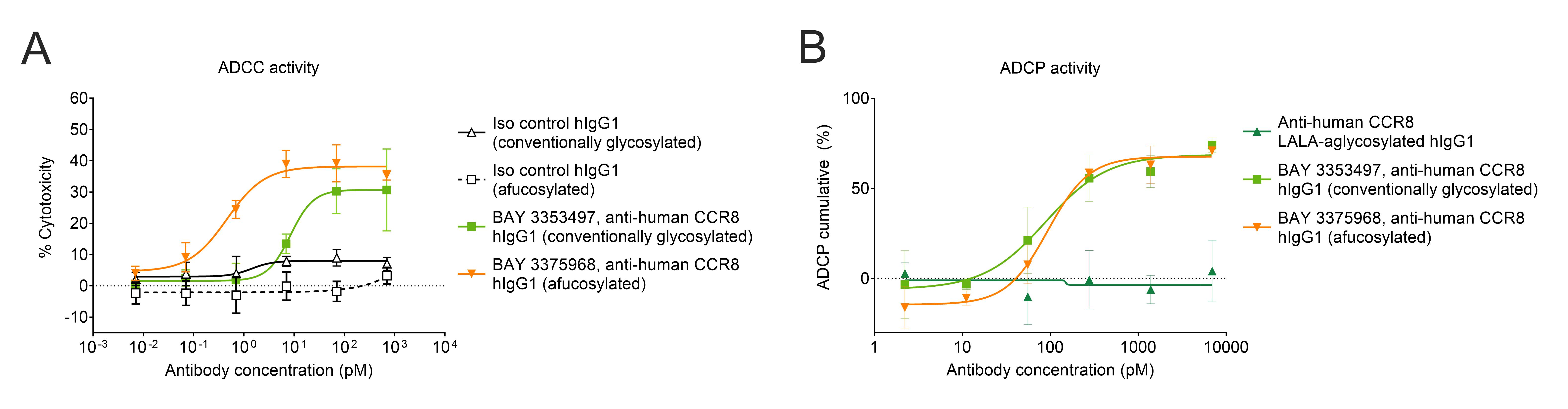

Supplement: Supplementary file 8 — Supplementary file8 (JPG 498 KB) [file 10238_2024_1362_MOESM8_ESM.jpg]
