# Supplementary material for: Selective depletion of tumor-infiltrating regulatory T cells with BAY 3375968, a novel Fc-optimized anti-CCR8 antibody
Source: Clin Exp Med. 2024 Jun 10;24(1):122. doi: 10.1007/s10238-024-01362-8 (PMC11164760; doi:10.1007/s10238-024-01362-8)
Supplement: Supplementary file 9 — Supplementary file9 (DOCX 315 KB) [file 10238_2024_1362_MOESM9_ESM.docx]

**SUPPLEMENTARY FIGURE LEGENDS AND TABLES**

**Supplementary Figure S1.** **Cellular binding of the anti-mouse CCR8 antibodies to mouse CCR8 as determined by flow cytometry.**

**A**. Cellular binding of anti-mouse CCR8 mIgG2a antibody and mIgG2a non-binding isotype control to murine CCR8-transfected HEK293 cells. Data are median MFI values from one representative experiment (n= 1).

**B**. Cellular binding of anti-mouse CCR8 hIgG1, anti-mouse CCR8 N297-aglycosylated hIgG1 antibodies and the hIgG1 non-binding isotype control to murine CCR8-transfected HEK293 cells. Data are median MFI values from one representative experiment (n= 1).

**C.** Cellular binding of anti-mouse CCR8 mIgG2a antibody and mIgG2a non-binding isotype control to mock-transfected HEK293 cells. Data are median MFI values from one representative experiment (n= 1).

**D.** Cellular binding of anti-mouse CCR8 hIgG1, anti-mouse CCR8 N297-aglycosylated hIgG1 antibodies and the hIgG1 non-binding isotype control to mock-transfected HEK293 cells. Data are median MFI values from one representative experiment (n= 1).

**Supplementary Figure S2. Tolerability and efficacy of anti-mouse CCR8 antibody treatments in mice, and the impact of co-depleting CD8+ T cells and CCR8+ Tregs on tumor growth inhibition.**

**A.** Relative body weight change of CT26 murine tumor-bearing mice treated with the anti-mouse CCR8 mIgG2a, hIgG1 antibodies, anti-mouse CCR8 N297A-aglycosylated hIgG1 antibody, and with the respective non-binding isotype controls (all at 10 mg/kg, Q3/4D, i.p., n= 10 mice/group). Q3/4D: every third or fourth day (twice weekly), i.p.: intraperitoneally.

**B.** Tumor growth in MC38 tumor bearing mice expressing diphtheria toxin receptor under the control of the CD8A promoter. Mice were treated with mIgG2a isotype control (10 mg/kg, Q3/4Dx4, i.p.) or anti-mouse CCR8 mIgG2a antibody (10 mg/kg, Q3/4Dx4, i.p.) as monotherapies or in combination with diphtheria toxin (0.015 mg/kg, Q3/4Dx4, i.p.) (n= 10 mice/group) to specifically remove CD8+ T cells. Black arrows indicate treatment days (days 8, 11, 15, and 18).

**Supplementary Figure S3.** **Dose-dependent depletion of CCR8+ Tregs from mouse tumors and its correlation with CD8+ T cell tumor infiltration and induction of IFNγ.**

**A.** Tumor growth inhibition in EMT6 tumor bearing mice treated with mIgG2a non-binding isotype control (10 mg/kg, Q3/4Dx3, i.p., n= 10 mice/group) or with an increasing concentration of anti-mouse CCR8 mIgG2a antibody (0.01, 0.1, 1, or 10 mg/kg, Q3/4Dx3, i.p., n= 10 mice/group). Black arrows indicate treatment days (days 7, 10, and 14). Treatment with anti-mouse CCR8 mIgG2a antibodies resulted in treatment/control (T/C) values of 0.33 for 10 mg/kg (p<0.001), 0.45 for 1 mg/kg (p<0.01), 0.90 for 01 mg/kg (p<0.5), and 1.12 for 0.01 mg/kg (p<0.4). Statistical analysis was performed using an ANOVA model with contrasts. Asterisks indicate statistical significance in comparison to isotype control (**p<0.01, ***p<0.001).

**B.** Tumor volumes in all treatment groups described in (A) at the end of the study on day 19 relative to tumor inoculation (n= 10 mice/group). Statistical analysis was performed using an ANOVA model with contrasts. Asterisks indicate statistical significance in comparison to isotype control (**p<0.01, ***p<0.001).

**C.** Relative body weight change of EMT6 tumor bearing mice treated as described in (A).

**D.–F.** Intratumoral (D) Tregs (CD4+ CD25+ FoxP3+) on day 11, corresponding to 24 h after second antibody treatment, and intratumoral (E) CD8+ T cells on day 19, at the end of the study, in EMT6 tumor lysates from mice described in (A) as determined by flow cytometry (n= 5/group). Statistical analysis was performed using an ANOVA model with contrasts. Asterisks indicate statistical significance in comparison to isotype control (*p<0.05, **p<0.01, ***p<0.001). F. Calculated ratio of intratumor CD8+ T cells to Tregs (CD45+ CD4+ CD25+ FoxP3+).

**G.–H.** IFNy concentrations in (G) tumors and (H) plasma of mice treated as described in (A), measured at the end of the study on day 19 and determined by ELISA (n= 5/group). Statistical analysis was performed using an ANOVA model with contrasts. Asterisks indicate statistical significance in comparison to isotype control (*p<0.05, **p<0.01).

**Supplementary Figure S4.** **PK/PD relationship of anti-mouse CCR8 mIgG2a antibody in mice using the EMT6 tumor model.**

Intratumoral CCR8+ Tregs (CD4+ CD25+ FoxP3+ CCR8+) over time in EMT6 tumor bearing mice treated with a single dose of mIgG2a non-binding isotype control (4 mg/kg, i.p.), or with increasing concentration of the anti-mouse CCR8 mIgG2a antibody (0.25, 1, or 4 mg/kg; i.p.) (n= 5 mice/group). Statistical analysis was performed using an ANOVA model with contrasts. *p<0.05, ***p<0.001 compared with the isotype control.

**Supplementary Figure S5. Combination of CCR8+ Treg depletion with anti-PD-1 immune checkpoint blockade results in enhanced anti-tumor activity.**

**A.** Intratumoral CD8+ T cell influx upon treatments of syngeneic mouse tumor models Hepa1-6, MC38, PANC02, H22, CT26 and MBT2 with anti-mouse CCR8 mIgG2a or hIgG1 antibodies and respective non-binding isotype controls, as measured by RNAseq (n= 8-10 mice/group).

**B.** Intratumoral *INFγ* expression upon treatments of syngeneic mouse tumor models Hepa1-6, MC38, PANC02, H22, CT26 and MBT2 with anti-mouse CCR8 mIgG2a or hIgG1 antibodies and respective non-binding isotype controls, as measured by RNAseq (n= 8-10 mice/group).

**C.** Efficacy of anti-mouse CCR8 mIgG2a antibody and anti-PD-1 antibody as monotherapy and in combination treatments using the syngeneic MBT2 murine tumor model. Tumor growth in mice treated with non-binding isotype controls, anti-mouse CCR8 mIgG2a antibody (10 mg/kg, Q3/4Dx5, i.p.), an anti-PD-1 antibody (10 mg/kg, Q3/4Dx5, i.p.), or the combination of anti-mouse CCR8 mIgG2a (10 mg/kg, Q3/4Dx5, i.p.) and the anti-PD-1 antibodies (10 mg/kg, Q3/4Dx5, i.p.). T/C values were for anti-mouse CCR8 mIgG2a antibody 0.5, for anti-PD-1 antibody 0.66 and for combination of both 0.15. Black arrows indicate treatment days (days 10, 13, 17, 20, 24) relative to tumor inoculation (n= 10 mice/group).

**D.** Efficacy of anti-mouse CCR8 mIgG2a antibody and anti-PD-1 antibody as monotherapy and in combination treatment in the syngeneic MB49 murine tumor model. Tumor growth in mice treated with vehicle (PBS), anti-mouse CCR8 mIgG2a antibody (10 mg/kg, Q3/4Dx4, i.p.), an anti-PD-1 antibody (10 mg/kg, Q3/4Dx4, i.p.), or the combination of anti-mouse CCR8 mIgG2a (10 mg/kg, Q3/4Dx4, i.p.) and the anti-PD-1 antibodies (10 mg/kg, Q3/4Dx4, i.p.). T/C values were for anti-mouse CCR8 mIgG2a antibody 0.37 (p<0.001), for anti-PD-1 antibody 0.63 (p<0.01) and for combination of both 0.16 (p<0.001). Statistical analysis was performed using an ANOVA model with contrasts. Asterisks and section signs indicate statistical significance in comparison to vehicle control and anti-PD-1 monotherapy groups, respectively (**p<0.01, ***,^§§§^p<0.001). Black arrows indicate treatment days (days 4, 7, 11, 15) relative to tumor inoculation (n= 10 mice/group).

**E.-F.** Intratumoral (E) Tregs and (F) ratio of CD8+ T cells to Tregs in MB49 tumors of mice treated as described in (D) and as determined by flow cytometry (n= 5/group). Tumors were collected at the end of the study on day 18. Statistical analysis was performed using an ANOVA model with contrasts. Asterisks, hashtags, and section signs indicate statistical significance in comparison to vehicle control, anti-mouse CCR8 mIgG2a monotherapy, and anti-PD-1 monotherapy, respectively (**,^##^p<0.01, *,^§^p<0.05).

**Supplementary Figure S6.** **Cellular binding of BAY 3375968 and other anti-human CCR8 antibodies to human CCR8 as determined by flow cytometry.**

**A.** Cellular binding of the anti-human CCR8 afucosylated hIgG1 antibody BAY 3375968, its conventionally glycosylated isoform BAY 3353497, LALA-aglycosylated anti-human CCR8 hIgG1 antibody and the respective non-binding isotype controls to human CCR8-transfected CHO cells. Data are median MFI values from one representative experiment (n= 1).

**B**. Cellular binding of the anti-human CCR8 afucosylated hIgG1 antibody BAY 3375968, its conventionally glycosylated isoform BAY 3353497, LALA-aglycosylated anti-human CCR8 hIgG1 antibody and the respective non-binding isotype controls to mock-transfected CHO cells. Data are median MFI values from one representative experiment (n= 1).

**Supplementary Figure S7: ADCC and ADCP activity of BAY 3375968 and other anti-human CCR8 antibodies.**

**A.** ADCC activity of afucosylated anti-human CCR8 hIgG1 antibody BAY 3375968, conventionally glycosylated anti-human CCR8 hIgG1 antibody BAY 3353497, afucosylated and conventionally glycosylated hIgG1 non-binding isotype controls, in co-culture of human CCR8-expressing HEK293 target cells and primary human NK cells as effector cells, at an E:T ratio of 4:1 (n= 3). Cytotoxicity was determined by measuring target cell apoptosis induction relative to the no antibody control at the 4 h co-culture time point.

**B**. ADCP activity (cumulative) of afucosylated anti-human CCR8 hIgG1 antibody BAY 3357968, conventionally glycosylated anti-human CCR8 hIgG1 antibody BAY 3353497, and LALA-aglycosylated anti-human CCR8 hIgG1 antibodies in co-culture of human CCR8-expressing HEK293 target cells and primary human M2 macrophages as effector cells, at E:T ratio of 10:1 (n= 3). The percentage of phagocytosed target cells was determined by measuring phagocytosis relative to no antibody control during the 24 h long co-culture period.

**Supplementary Table 1.** Antibodies used in this study.

| **Antibody** | | **Isotype** | **Description** | **Provider** |
| --- | --- | --- | --- | --- |
| Non-binding isotype control antibodies | Isotype control | mIgG2a | Non-binding isotype control | Bayer AG |
|  | Isotype control | hIgG1 | Non-binding isotype control (conventional glycosylation) | Bayer AG |
|  | Isotype control | hIgG1 | Non-binding isotype control  (N297A-aglycosylated) | Bayer AG |
|  | Isotype control  (afucosylated) | hIgG1 (afuco) | Non-binding isotype control  (afucosylated) | Bayer AG |
|  | Isotype control | mIgG1 | Non-binding isotype control for anti-PD-L1 antibody | Bayer AG |
|  | Isotype control | rIgG2a | Non-binding isotype control for anti-PD-1 antibody | BioXCell, Clone 2A3 |
|  | Anti-mouse CCR8 mIgG2a antibody | mIgG2a | Anti-mouse CCR8 antibody, human VH/VL chimerized to mouse IgG2a | Bayer AG |
|  | Anti-mouse CCR8 hIgG1 antibody | hIgG1 | Anti-mouse CCR8 antibody (conventional glycosylation) | Bayer AG |
|  | Anti-mouse CCR8 hIgG1 aglyco antibody | hIgG1 | Anti-mouse CCR8 antibody, N297A-aglycosylated variant of hIgG1 antibody | Bayer AG |
|  | Anti-human CCR8 BAY 3375968 | hIgG1 | Anti-human CCR8 antibody (afucosylated) | Bayer AG |
| Target-specific antibodies | Anti-human CCR8 BAY 3353497 | hIgG1 | Anti-human CCR8 antibody (wild type with conventional glycosylation) | Bayer AG |
|  | Anti-human CCR8 Fc-silenced antibody | hIgG1 | Anti-human CCR8 antibody, LALA-aglycosylated (L234A, L235A, N297A) variant of BAY 3375968 | Bayer AG |
|  | Anti-PD1 antibody | rIgG2a | Anti-mouse PD-1 antibody | BioXCell, BE0146 |
|  | Anti-PD-L1 antibody | mIgG1 | Anti-mouse PD-L1 antibody | Bayer AG |
|  | Mogamulizumab | hIgG1 | Anti-CCR4 antibody (afucosylated) | Kyorin Pharmaceuticals Co |
|  | Anti-HLA antibody | hIgG1 | Anti-human HLA complex I antibody | Bayer AG |
| CCR8, C-C motif chemokine receptor 8; hIgG, human immunoglobulin gamma; mIgG, mouse immunoglobulin gamma; PD-1, programmed cell death protein 1; PD-L1, programmed death ligand 1; rIgG, rat immunoglobulin gamma; VH/VL, variable fragment heavy chain/variable fragment light chain; HLA-I: human leukocyte antigen system class I | | | | |

**Supplementary Table S2.** Binding of anti-mouse CCR8 antibodies to mouse FcγR variants and mouse FcRn as determined by SPR using a Biacore T200 instrument.

| **Name** | **KD [nM]** | | |
| --- | --- | --- | --- |
|  | **Anti-mouse CCR8 Ab mIgG2a** | **Anti-mouse CCR8 Ab hIgG1** | **Anti-mouse CCR8 Ab hIgG1 aglyco** |
| Mouse FcγRI/CD64 | 98 | 590 | 6.700 |
| Mouse FcγRIIB/CD32b | 12.960 | 4.070 | no binding |
| Mouse FcγRIII/CD16 | 9.086 | no binding | no binding |
| Mouse FcγRIV/CD16-2 | 610 | 1.062 | low binding^#^ |
| Mouse FcRn | 246 | 10* | 7* |

* Approximation due to slight unspecific binding

^#^ Low binding response at highest concentration of 25 µM, not quantifiable

**Supplementary Table S3.** Plasma concentration of anti-mouse CCR8 antibodies in CT26 tumor bearing mice (as described in Fig. 2C).

| **Timepoint** | **Anti-mouse CCR8 Ab hIgG1-aglyco**  **[µg/L, GM ± GSD]** | **Anti-mouse CCR8 Ab hIgG1**  **[µg/L, GM ± GSD]** | **Anti-mouse CCR8 Ab mIgG2a**  **[µg/L, GM ± GSD]** |
| --- | --- | --- | --- |
| 24 h | 106,713.2 ± 1.2 | 81,650.8 ± 1.3 | 123,794.5 ± 1.3 |
| 96 h | 82,852.5 ± 1.2 | 13,179.5 ± 1.2 | 67,407.7 ± 1.1 |
| 144 h | 63,417.6 ± 1.1 | 889.2 ± 10.4 | 39,499.8 ± 1.2 |
| n = 3/timepoint. GM, geometric mean; GSD, geometric standard deviation | | | |

**Supplementary Table S4.** Plasma concentration of the anti-mouse CCR8 mIgG2a antibody in EMT6 tumor-bearing mice over time after single dose treatment (as described in Supplementary Figure S4).

| **Time point** | **Concentration of anti-mouse CCR8 mIgG2a antibody in plasma (µg/L, GM ± GSD)** | | |
| --- | --- | --- | --- |
|  | **0.25 mg/kg** | **1 mg/kg** | **4 mg/kg** |
| 2 h | 361.6  ± 1.2 | 4,006  ± 1.4 | 6717.6  ± 3.0 |
| 24 h | 276.9  ± 1.2 ^a^ | 1,470.4  ±1.4 | 7,677.1  ± 1.2 |
| 48 h | 273.4  ±1.3 ^a^ | 1,041.7  ± 1.4 ^a^ | 6,032.3  ±1.0 ^b^ |
| 120 h | 84.5  ±1.2 | 201.4  ± 4.0 ^a^ | 1,439.1  ± 1.8 |
| 192 h | 70.3  ±1.2 ^c^ | 54.4  ± 2.0 ^a^ | 88.3  ± 3.4 ^a^ |
| 336 h | 43.2  ±1.7 ^b^ | 45.4  ± 1.5 ^c^ | 161.7  ± 1.0 ^d^ |
| n= 5/time point unless indicated otherwise. GM, geometric mean; GSD, geometric standard deviation; LLQ, lower limit of quantification  ^a^ n= 4  ^b^ n= 2  ^c^ n= 3  ^d^ n= 1 | | | |

**Supplementary Table S5.** Antitumor efficacy and intratumoral Treg depletion across 23 different syngeneic mouse tumor models treated with 10 mg/kg anti-mouse CCR8 mIgG2a or hIgG1 antibodies.

|  | **Mouse Strain** | **Cancer Cell Line** | **Tissue of origin** | **Treatment/Control (T/C) ratio ^a^** | **Treg depletion (%) ^b^** |
| --- | --- | --- | --- | --- | --- |
| Good- and medium-responding models | BALBc | CT26 | Colon cancer | 0.18 | 82.3 |
|  | C57BLJ | F9 | Testicular cancer | 0.21 | 73.2 |
|  | C57BLJ | MC38 | Colon cancer | 0.23 | 68.2 |
|  | BALBc | EMT-6 | Breast cancer | 0.28 | 61.6 |
|  | BALBc | H22 | Liver cancer | 0.30 | n.d. |
|  | C57BLJ | B16F10-OVA | Melanoma | 0.37 | 59.0 |
|  | C57BLN | MB49 | Bladder cancer | 0.37 | 45–75 |
|  | C3HHE | MBT2 | Bladder cancer | 0.40 | n.d. |
|  | BALBc | 4T1 | Breast cancer | 0.42 | n.d. |
|  | C57BLJ | Hepa1-6 | Liver cancer | 0.45 | n.d. |
|  | BALBc | A20 | Lymphoma | 0.46 | n.d. |
|  | BALBc | Colon26 | Colon cancer | 0.50 | n.d. |
|  | C57BLJ | PANC02 | Pancreatic cancer | 0.51 | n.d. |
| Low- and non-responding models | DBA2 | KLN205 | Lung cancer | 0.79 | n.d. |
|  | BALBc | RENCA | Renal cancer | 0.81 | n.d. |
|  | C57BLJ | LL2 | Lung cancer | 0.85 | n.d. |
|  | C57BLJ | EG7-OVA | Lymphoma | 0.86 | n.d. |
|  | BALBc | J558 | Plasmacytoma | 0.89 | n.d. |
|  | C57BLJ | B16F10 | Melanoma | 0.95 | 7.5 |
|  | C57BLJ | RM1 | Prostate cancer | 0.99 | n.d. |
|  | C57BLJ | EL4 | Lymphoma | 1.05 | n.d. |
|  | C57BLJ | B16BL6 | Melanoma | 1.06 | n.d. |
|  | BALBc | WEHI-164 | Fibrosarcoma | 1.16 | n.d. |
| n.d., not determined.  ^a^ as determined from tumor volumes at the end of the study. Good<0.4; medium 0.4–0.7; low>0.7.  ^b^ as determined by flow cytometry 24 h after 2nd treatment, otherwise at the end of the study | | | | | |

**Supplementary Table S6.** Binding of anti-human CCR8 antibodies to human FcγR variants and human FcRn as determined by SPR using a Biacore T200 instrument.

| **Fc receptor** | | BAY 3375968  (Anti-human CCR8 afucosylated hIgG1) | BAY 3353497  (Anti-human CCR8 conventionally glycosylated hIgG1) |
| --- | --- | --- | --- |
| Human | FcγRI/CD64 | 98 | 70 |
|  | FcγRIIA/CD32a, isoform R131 | 11,000 | 6,900 |
|  | FcγRIIB/C/CD32b/c | > 25,000 ^b^ | 19,000 ^a^ |
|  | FcγRIIIB/CD16b | 2,300 | 18,000 |
|  | FcγRIIIA/CD16a, isoform V158 | 220 | 2,700 |
|  | FcγRIIIA/CD16a, isoform F158 | 230 | 5,100 |
|  | FcRn | 50 | 62 |
| FcRn, neonatal Fc receptor  ^a^Values as a rough approximation only, as the highest concentration is 25,000 nM and saturation might not be reached  ^b^KD >25,000 nM: fitted value outside of saturation curve | | | |

**Supplementary Table S7.** Comparison of ADCC activity of anti-human CCR8 hIgG1 antibodies BAY 3357968 and BAY 3353497 in assays using human NK92v or primary human NK cells as effector cells.

| **Antibody** | **NK92v cell hADCC**  **(n=3)** | | | **Primary NK cell hADCC**  **(n=2)** | | |
| --- | --- | --- | --- | --- | --- | --- |
|  | EC50 potency (pM) | Max response (%) | EC50 potency (pM) | | | Max response (%) |
| Anti-human CCR8,  BAY 3353497  (Conventionally glycosylated) | 34.16 | 28.37 | | | 8.12 | 26.30 |
| Anti-human CCR8,  BAY 3357968  (Afucosylated) | 0.35 | 40.91 | | | 0.48 | 32.77 |
